# Inflammatory factors collaboratively link *Helicobacter pylori*-induced gastritis to gastric cancer

**DOI:** 10.3389/fimmu.2025.1628543

**Published:** 2025-11-25

**Authors:** Mingze Zhang, Ade Su, Houji Song, Siyu Zhang, Yuan Deng, Wutang Jing, Jin Guo, Weipeng Zhan, Yuntao Ma, Ming Hu

**Affiliations:** 1 Department of General Surgery, Gansu Provincial Hospital, Lanzhou, China; 2 The Second Clinical School of Medicine, Lanzhou University, Lanzhou, China; 3 NHC Key Laboratory of Diagnosis and Therapy of Gastrointestinal Tumor, Gansu Provincial Hospital, Lanzhou, China; 4 Key Laboratory of Molecular Diagnostics and Precision Medicine for Surgical Oncology in Gansu Province, Gansu Provincial Hospital, Lanzhou, China

**Keywords:** gastritis, gastric cancer, inflammatory factors, tumor micro environment, *Helicobacter pylori*

## Abstract

Long-term inflammatory reaction may promote gastric cancer initiation and development through multiple mechanisms. Recent studies have demonstrated that inflammatory mediators play a crucial role in the transition from gastritis to gastric cancer. Pro-inflammatory cytokines, chemokines, and other signaling molecules interact and synergistically regulate gastric epithelial cell proliferation, apoptosis, migration, and invasiveness, thereby promoting tumorigenesis. Specifically, interleukins activate immune cells, induce the secretion of inflammatory mediators, and maintain local immune responses; however, in the context of cancer, they exhibit a dual role by both enhancing anti-tumor immunity and driving tumor progression. Tumor necrosis factor amplifies immune responses by stimulating the production of pro-inflammatory cytokines, yet excessive or chronic Tumor necrosis factor activity is a hallmark of autoimmune diseases. Interferons initiate antiviral responses, modulate immune cell functions, and influence the inflammatory cascade. Chemokines primarily mediate the recruitment of immune cells to sites of infection, inflammation, or injury, but also play key roles in immune evasion and tumor immune regulation. This review summarizes the cooperative roles of these inflammatory mediators in the progression from gastritis to gastric cancer and discusses their potential as therapeutic targets. A better understanding of these mechanisms may facilitate the development of novel strategies for the prevention and treatment of gastric cancer.

## 1 Introduction

### 1.1 Inflammation and tumorigenesis

In 1863, Rudolf Virchow first proposed the connection between inflammation and cancer, suggesting that certain stimuli, along with the tissue damage and inflammation they induce, can drive cell proliferation (1, 2). The inflammatory cells and cytokines present in the TME play a crucial role in promoting tumor growth, metastasis, and modulating immune responses (3). This concept has evolved over time, and decades of research have provided further validation of this link. Chronic inflammation is considered a marker of cancer (4). Mutations contribute to tumorigenesis; however, in the majority of cases (>90%), cancer development is closely linked to chronic inflammation in some form (5).

The relationship between inflammation and tumorigenesis is both complex and deeply interconnected, with chronic inflammation widely regarded as a key factor driving tumor initiation and progression across various cancers. Inflammatory processes, whether infectious—such as *H. pylori*-induced gastritis (6) or hepatitis B virus-related chronic hepatitis (7)—or non-infectious (8), including autoimmune diseases and chronic tissue damage caused by environmental factors, contribute to tumorigenesis through multifaceted mechanisms (9–12). Chronic inflammation is often characterized by repeated cycles of tissue injury and repair, leading to accelerated cell proliferation, genetic mutation accumulation, disrupted signaling pathways, and diminished immune surveillance, collectively creating a conducive environment for tumor development (9, 13).

In the context of chronic inflammation, inflammatory cells such as macrophages, neutrophils, and lymphocytes release significant amounts of pro-inflammatory cytokines (e.g., IL-6, TNF-α, IL-1β), chemokines, and reactive oxygen species (ROS) or reactive nitrogen species (RNS). These mediators not only induce direct DNA damage (14) but also lead to epigenetic alterations (15, 16) that silence tumor suppressor genes or activate oncogenes. Additionally, pro-inflammatory signals activate critical intracellular pathways such as NF-κB and STAT3, which drive abnormal cell proliferation, inhibit apoptosis, and enhance the invasive and metastatic capabilities of cells (17, 18). Accumulated ROS and RNS further impair DNA repair mechanisms, heightening genomic instability and fostering conditions that facilitate the emergence of cancer cells (19, 20).

Beyond cellular effects, inflammation profoundly influences tumorigenesis by shaping the TME (21). Chronic inflammation drives ECM remodeling paving the way for tumor cell invasion and metastasis (22). Furthermore, pro-angiogenic factors like VEGF (23) secreted within the inflammatory milieu significantly promote angiogenesis, supplying tumors with essential nutrients and oxygen while enabling cancer cells to enter the circulatory system (22, 24). Chronic inflammation also weakens immune surveillance. For instance, TAMs (25) and MDSCs (26), which accumulate in inflammatory conditions, secrete immunosuppressive cytokine that dampen the activity of effector T cells, thereby aiding tumor cells in evading immune responses.

The effects of inflammation on tumorigenesis vary across tissue types and inflammation forms. Chronic inflammation is notably linked to specific cancers, such as colorectal cancer associated with chronic ulcerative colitis (27) and hepatocellular carcinoma linked to chronic hepatitis (28). Compared to acute inflammation, which may transiently activate immune defenses, chronic inflammation exerts more subtle yet persistent effects, including genomic instability, localized immune suppression, and profound alterations to the TME, thereby amplifying tumorigenic potential.In fact, not all chronic inflammatory diseases increase the risk of cancer. Some of these diseases, such as psoriasis, can even reduce the risk of cancer (29).

In conclusion, inflammation serves as a “double-edged sword” in tumorigenesis. While acute inflammation may bolster immune surveillance and eliminate abnormal cells, chronic inflammation promotes genetic mutations, activates oncogenic pathways, suppresses immune defenses, and reconfigures the TME, thereby facilitating cancer initiation and progression. Elucidating the mechanisms linking chronic inflammation to tumorigenesis will deepen our understanding of cancer biology and support the development of innovative anti-inflammatory and anticancer therapies, paving the way for more effective and personalized treatment strategies.

### 1.2 Inflammation and tumorigenesis

Inflammatory factors are a class of cytokines, chemical substances, or small molecules secreted by immune cells, epithelial cells, and other tissue cells during the inflammatory response (30). These factors play a critical role in regulating the immune system, promoting tissue repair, and maintaining homeostasis. However, the excessive or prolonged activation of inflammatory factors may lead to chronic inflammation, which can trigger a variety of diseases, including autoimmune diseases (31), cardiovascular diseases (32), and cancer (33).

**Figure 1:**
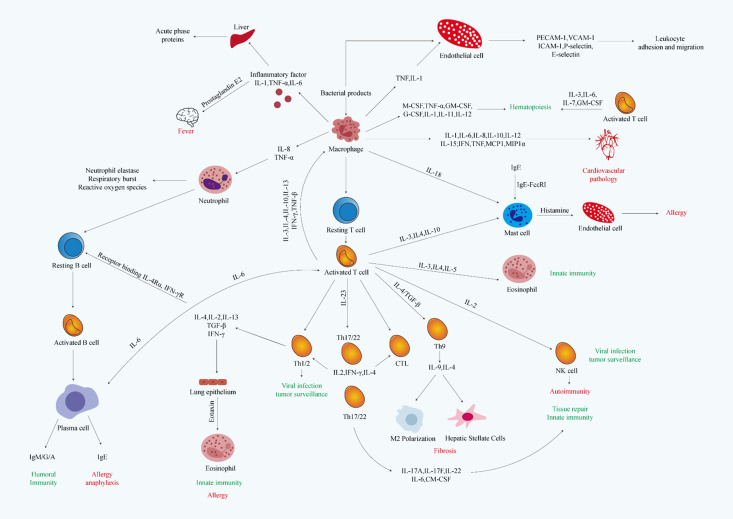
Schematic representation of the dynamic regulatory network of inflammatory factor secretion, cellular targeting effects, and associated molecular mechanisms. The figure was adapted from Thermo Fisher (https://www.thermofisher.cn/). Red text indicates system-related pathologies, green text denotes biological or pathological processes, and black text represents structural or molecular entities.

Based on their function and chemical properties, inflammatory factors can be classified into several categories: Pro-inflammatory factors (34) enhance the inflammatory response by activating pro-inflammatory signaling pathways, resulting in tissue damage and abnormal cell proliferation. Second, anti-inflammatory factors (34) play a key role in maintaining the balance of the inflammatory response by inhibiting the production of pro-inflammatory factors and reducing tissue damage. In addition, chemokines (34) primarily function to recruit immune cells to the site of inflammation, thereby expanding the scope of the inflammatory response. The functions of inflammatory factors and their communication network are shown in **Table 1** and **Figure 1**.

**Table 1:** Inflammatory factors and their functions.

Classify	Cytokines	Source	Receptor	Target cell	Key features
IL	IL-1	Macrophages, B cell, dendritic cell	CD121a	B cells, NK cell, T cell	Pyrogenic (34, 35), pro-inflammatory (36), proliferative and differentiated (37)
	IL-2	Th1 cell	CD25	Activated T cells and B cell, NK cell	Adaptive immunity (38), cell proliferation (39), activated T cell, NK cell function (40)
	IL-4	Th cell	CD124	B cell, T cell, macrophage	Adaptive immunity (41), B cell and cytotoxic T cell proliferation (42), enhances MHC class II expression (43), and stimulates IgG and IgE production (44)
	IL-6	Th cell, macrophage, fibroblast	CD126, 130	B cell, plasma cell	pro-inflammatory (45), B cell differentiation (46)
	IL-10	T cell, B cell, macrophage	CDw210	B cell, macrophage	Anti-inflammatory (47), Inhibits cytokine production and monocyte function (48) (49)
	IL-12	T cell, macrophage, monocyte	CD212	NK cell, macrophage, tumor cell	Pro-inflammatory (50), Activation of NK cell, phagocytic cell activation (51), endotoxin shock (52), tumor cytotoxicity (53), cachexia (54)
	IL-17	Th17 cell	IL-17R	Monocyte, neutrophil	Monocytes and neutrophils are recruited to the site of infection (55)
	IL-18	Macrophage, dendritic cell, and epithelial cell	CD218a (IL-18Ra)	Monocyte and T cell	Recruit monocytes and T lymphocytes (56). In combination with IL-12, it induces IFN-γ production and inhibits angiogenesis (57).
TNF	TNF-α	Macrophage	CD120a, b	Macrophage	Pro-inflammatory (58), Phagocytic cell activation (59), endotoxin shock (60)
	TNF-β	T cell	CD120a, b	Macrophage, tumor cell	Pro-inflammatory (61), Chemotactic, phagocytosis, tumor suppression, induction of other cytokines (62)
IFN	IFN-α	macrophage, neutrophil, and some somatic cell	CD118 (IFNAR1, IFNAR2)	extensive	Pro-inflammatory (63), Antiviral (64)
	IFN-β	fibroblast	CD118 (IFNAR1, IFNAR2)	extensive	Pro-inflammatory (65), Antiviral (66), antiproliferative (67)
	IFN-γ	T cell and NK cell	CDw119 (IFNG R1)	extensive	Pro-inflammatory (68), Antiviral (69), macrophage activation (70), enhanced neutrophil and monocyte function (71) and expression of MHC-I and -II on cells (72)
Chenokines	CCL2	Endothelial cell, monocyte, fibroblast	CCR2, CCR4	Basophil, monocyte, T cell, dendritic cell	Induces chemotaxis (73), regulates macrophage activity (74), and regulates cytokine production (75)
	CCL3	Monocyte, neutrophil, fibroblast, and dendritic cell	CCR1, CCR4, CCR5	Eosinophil, monocyte, T cell, dendritic cell	Induces various pro-inflammatory activities, such as leukocyte chemotaxis (76). Granulomas, asthma, T1D, and key inflammatory mediators in other autoimmune diseases (77)
	CCL5	T cell, monocyte, NK cell,	CCR1, CCR3, CCR4, CCR5	Basophil, eosinophil, monocyte, T cell, dendritic cell	Promotes apoptosis (78), antiviral (79), tumor development (80), and plays a role in insulin secretion of pancreatic islet cells by activating GPR75 (81)
	CXCL8(IL-8)	Neutrophil, endothelial cell, fibroblast	CXCR1, CXCR2	Neutrophil, basophil	Recruitment and activation of neutrophils to sites of inflammation (82), tissue damage (83), fibrosis (84), angiogenesis (85), and tumorigenesis (86)
	CXCL10	Monocyte, endothelial cell, fibroblast	CXCR3	Monocyte, T cell, NK cell	Chemotactic activity (87), induces apoptosis (88), regulates cell growth and proliferation, and tumor formation (89)
	CXCL12	Stromal cell	CXCR4, CXCR7	All cell types	It plays a key role in the pathological process of some diseases such as inflammation, tumor formation and metastasis, pathogen infection, wound repair, etc. (90) (91)
	XCL1	T cell, NK cell	XCR1	T cell	Chemotactic activity (92), which contributes to the development of T cells (93)
	CX3CL1	Endothelial cell, neuronal cell	CX3CR1	Monocyte, T cell, NK cell	Chemotactic activity (94), immune response (95), inflammation (96), cell adhesion (97)

Inflammatory factors play a central role in the link between inflammation and cancer through various mechanisms. In GC, inflammatory factors contribute to tumorigenesis by activating signaling pathways, reshaping the TME, and suppressing immune surveillance, thus driving the entire process from early tumor formation to late-stage metastasis (98). A comprehensive understanding of the function and regulatory mechanisms of inflammatory factors will not only help elucidate the pathogenesis of GC but also provide novel insights into the development of targeted anti-inflammatory cancer therapies, laying a theoretical foundation for personalized treatment strategies.

### 1.3 Gastritis and GC

Gastritis broadly refers to inflammatory or reactive injury of the gastric mucosa with diverse etiologies (e.g., *H. pylori*, autoimmune atrophic gastritis, bile-reflux/chemical injury, eosinophilic or lymphocytic gastritis). Clinically, it is important to distinguish reactive/chemical injury from leukocyte-predominant inflammatory gastritis and to record acute versus chronic patterns and anatomic distribution (antrum-predominant, corpus-predominant, or pangastritis), which in turn influence mechanisms and risks of progression from chronic inflammation to cancer (99, 100). GC is a significant global health issue, often resulting from a multifactorial process involving genetic, environmental, and microbial factors (101, 102).

When gastritis becomes chronic, it can lead to progressive damage of the stomach lining, starting with atrophy (thinning of the gastric mucosa), followed by metaplasia (the transformation of normal cells into abnormal ones) and dysplasia (abnormal cell growth) (99). These changes are considered precursors to GC. Persistent inflammation can also lead to the accumulation of genetic mutations, disruption of normal cell signaling pathways, and the activation of pro-inflammatory factors, all of which contribute to the development of cancer. If left untreated, this chronic inflammatory process can eventually promote the transformation of normal gastric cells into malignant cancer cells, resulting in GC.

## 2 ILs in inflammation and cancer

ILs play a central role in inflammation by regulating the immune response and the inflammatory response (103, 104). By promoting the activation of immune cells, secreting pro-inflammatory factors, and maintaining local immune responses, they are involved in acute and chronic inflammatory processes. However, persistent or excess expression of ILs can lead to chronic inflammation and increase the risk of diseases like infectious diseases (105), cardiovascular diseases (106) and cancer (107).

In cancer, the role of ILs is even more complex. ILs can both enhance tumor immunity by modulating immune cell function in the TME (108) and drive tumor progression by promoting immune escape and tumor cell growth (109). Thus, the role of ILs in cancer is a dual one, both protective and potentially aggravating. The specific mechanism of IL in GC and gastritis is detailed in **Table 2**.

**Table 2:** The mechanism of IL in gastritis and GC.

Cytokines	Brief biological mechanism in gastritis	Brief biological mechanism in GC
IL-1	IL-1β Suppresses Gastrin via Primary Cilia and Induces Antral Hyperplasia, leading to gastritis (110) IL-1β play a role in chronic inflammation of the gastric mucosa in *H. pylori* infection with functional dyspepsia patients (111) ETS1 synergizes with IL-1 through the NF-κB signaling pathway for gastritis (112)	pylori infection, IL-1β is highly expressed that result in gastric acid inhibition, GC -related gene methylations and disfunctions, angiogenesis (113) IL-1 Up-regulates MicroRNA 135b to Promote Inflammation-Associated Gastric Carcinogenesis in Mice (114) IL-1β-associated NNT acetylation orchestrates iron-sulfur cluster maintenance and cancer immunotherapy resistance (115)
IL-2	Astaxanthin slows down gastritis of *H. pylori* infection by enhancing IL-2 secretion (116)	Tumor-infiltrating mast cells stimulate ICOS regulatory T cells through an IL-33 and IL-2 axis to promote GC progression (117)
IL-4	berberine activated IL-4-STAT6 signaling pathway *in vivo* and *in vitro* when *H. pylori* infection and presented anti-inflammatory activities (118) IL-4 addresses gastric inflammation by stimulating gastric D cells to release somatostatin (119)	Protoberberine alkaloids have demonstrated therapeutic effects on chronic atrophic gastritis and GC by activating IL-4/STAT6 pathway (120) IL-4 inhibited proliferation of HTB-135 GC cells by down-regulating G0-G1 cell cycle nuclear-regulating factors (121)
IL-6	Serum exosomes of chronic gastritis patients infected with *H. pylori* mediate IL-1α expression via IL-6 trans-signalling in gastric epithelial cells (122) Lactobacillus plantarum ZJ316 significantly reduces IFN-γ and IL-6 levels, increases IL-10 levels, repairs mucosal damage, and has preventive and therapeutic effects on *H. pylori* -induced gastritis (123) Weierning tablet reduces the mRNA level of IL-6 and thus improves gastritis (124) YJHD alleviated NLRP3 inflammasome formation and pyroptosis of epithelial cells in Chronic atrophic gastritis, potentially through the inactivation of IL-6/STAT3 pathways (125) *H pylori* gastritis is associated with increased gastric mucosal production of TNF alpha and IL-6 (126) *H. pylori* infection results in a local increase in ILs-6 receptor associated with high-grade mucosal inflammation (127)	*H. pylori* Activates IL-6-STAT3 Signaling in Human GC Cells: Potential Roles for ROS (128) IL-6 mediates epithelial-stromal interactions and promotes gastric tumorigenesis (129) RBMS1 promotes GC metastasis through autocrine IL-6/JAK2/STAT3 signaling (130) Berberine inhibits GC development and progression by regulating the JAK2/STAT3 pathway and downregulating IL-6 (131) VPS35 promotes GC progression through integrin/FAK/SRC signalling-mediated IL-6/STAT3 pathway activation in a YAP-dependent manner (132) MFGE8 promotes GC progression by activating the IL-6/JAK/STAT3 signaling (133)
IL-8	IL-8 upregulates the inflammatory response to *H. pylori* infection and plays an important role in cell proliferation and gastric mucosal injury (134) IL-8 may play an important role in neutrophil transport from mucosal blood vessels to gastric epithelium and may be involved in regulating *H. pylori* gastritis (135) astaxanthin inhibits *H. pylori*-induced ROS-mediated IL-8 expression by activating PPAR-γ and catalase in gastric epithelial cells (136) *H. pylori*-derived OMVs may aid the development of various gastric diseases by inducing IL-8 production and NF-κB activation (137) α-LA may prevent the development of *H. pylori*-associated gastric diseases by decreasing ROS-mediated IL-8 expression in gastric epithelial cells (138)	*H. pylori* with trx1 high expression promotes gastric diseases via upregulating the IL23A/NF-κB/IL8 pathway (139) CAFs-derived IL-8 plays important roles in chemoresistance, immunosuppression, and lymph node metastasis of GC (140) FAK/IL-8 axis promotes the proliferation and migration of GC cells (141) Cancer-Associated Fibroblast-Derived IL-8 Upregulates PD-L1 Expression in GC Through the NF-κB Pathway (142) Tumor-derived IL-8 facilitates lymph node metastasis of GC via PD-1 up-regulation in CD8 T cells (143)
IL-10	Yangyin Huowei mixture alleviates chronic atrophic gastritis by inhibiting the IL-10/JAK1/STAT3 pathway (144) *H. pylori* controls NLRP3 expression by regulating hsa-miR-223-3p and IL-10 in cultured and primary human immune cells (145) Regulatory dendritic cells produce IF-10 to protect against autoimmune gastritis in mice (146)	Gastric tumorigenesis induced by combining *H. pylori* infection and chronic alcohol through IL-10 inhibition (147) Gut microbiome-derived butyrate inhibits the immunosuppressive factors PD-L1 and IL-10 in TAMs in GC (148) IL−10 secreted by cancer−associated macrophages regulates proliferation and invasion in GC cells via c−Met/STAT3 signaling (149)
IL-12	PAR1 inhibits IRF5 and IL-12 secreted by macrophages, and the host inhibits mucosal Th1 and Th17 responses to *H. pylori* infection through this mechanism (150)	IL-12 treatment reduces tumor growth and modulates the expression of CASKA and MIR-203 in athymic mice bearing tumors induced by the HGC-27 GC cell line (151)
IL-17	*H. pylori* activate NF-κB signaling through CagA, thereby inducing IL-17A expression in FOXP3 T cells, leading to gastritis (152) IL-17 produces T cells capable of inducing severe autoimmune gastritis (153) IL-17 expression showed a significant increase with the severity of chronic gastritis (154) IL-17 induces IL-8 secretion by activating the ERK 1/2 MAP kinase pathway, and the released IL-8 attracts neutrophils to promote gastritis (155)	IL-17RA signaling activates a protective pathway to prevent excessive inflammation and reduces the risk of stomach cancer (156) Tumor-associated neutrophils induce EMT by IL-17a to promote migration and invasion in GC cells (157) IL-17B signaling in IL-17RB directly promotes cancer cell survival, proliferation, and migration, and induces resistance to conventional chemotherapeutic agents (158) LCN2 Mediated by IL-17 Affects the Proliferation, Migration, Invasion and Cell Cycle of GC Cells by Targeting SLPI (159)
IL-18	IL-18, and possibly CD14 receptor signalling pathway, may be involved in macrophage activation and subsequent IL-8 and IL-1 beta release, involved in gastritis response to *H. pylori* infection (160) IL-18 may have an important role in promoting gastric Th1 responses in *H. pylori* infection (161) The cytokine IL-18 induces production of IFN-γ by activated T lymphocytes and promotes a Th1 profile, causing chronic active gastritis (162)	Eupafolin hinders cross-talk between GC cells and cancer-associated fibroblasts by abrogating the IL18/IL18RAP signaling axis (163) Inflammasome Adaptor ASC Suppresses Apoptosis of GC Cells by an IL18-Mediated Inflammation-Independent Mechanism (164) IL-18 produced by gastric epithelial cells protects against pre-neoplastic lesions in *H. pylori* infection in mice (165)
IL-23	IL-23 was released in the presence of *H. pylori* from the inflamed gastric mucosa, which was positively correlated with neutrophil and monocyte infiltration (166) IL-23 plays a role in the activation of the immune response and induction of gastritis in response to *H. pylori* by contributing to the control of infection and severity of gastritis (167) Upregulation of IL-23 occurs early in the host response to *H. pylori* and may contribute to the severity of induced gastric lesions (168) A role for RUNX3 in inflammation-induced expression of IL23A in gastric epithelial cells (169)	IL-23 promotes the migration and invasion of GC cells by inducing epithelial-to-mesenchymal transition via the STAT3 pathway (170) IL-23A can promoted GC cells growth by inducing the secretion of IL-17A in TME (171) IL23 receptor, as a key cytokine receptor gene in the important inflammatory IL-17/IL-23 axis, may contribute to GC predisposition (172) IL-8 and IL-23 induced an inflammatory response and leading to apoptosis, which can lead to carcinogenesis (173)

## 3 ILs

### 3.1 IL-1

IL-1 is a pivotal cytokine produced by various cell types, including monocytes, macrophages, and fibroblasts, primarily in two isoforms: IL-1α and IL-1β (174). We will focus primarily on IL-1β, IL-1α, and IL-1β, although the IL-1 family also includes the disease-associated cytokines IL-18, IL-33, and IL-36 (175). It serves as a central mediator in the immune and inflammatory responses, regulating immune activity (176), enhancing inflammation (177), and influencing cellular proliferation and tissue repair through the activation of multiple signaling pathways (178, 179). The involvement of IL-1 in gastritis (180), GC (115), and the TME (181) is extensive and multifaceted, playing a significant role in the pathogenesis and progression of these conditions.

#### 3.1.1 Role of IL-1 in gastritis and GC

IL-1 is a critical mediator in the onset and progression of gastritis, especially in chronic forms, where elevated IL-1 levels amplify inflammation (182). Through activation of NF-κB, IL-1 induces the release of pro-inflammatory cytokines such as TNF-α and IL-6, exacerbating the inflammatory response (106, 183). During *H. pylori* infection, IL-1 promotes immune cell infiltration and gastric epithelium injury, which may exacerbate lesions and contribute to disease progression (184).

IL-1 promotes tumor growth and metastasis through a variety of mechanisms and plays an important role in GC. Such as NF-κB pathway, thereby promoting cell proliferation, survival, and metastasis (185). IL-1 also alters the TME by upregulating immune suppressive cells like T cells (186) and M2 macrophages (187), which reduces the immune response against tumors and promotes tumor growth.

#### 3.1.2 The role of IL-1 in the TME

In both gastritis and GC, IL-1 plays a key role in the tolerance of the immune system. In gastritis, IL-1 promotes immune responses, but if dysregulated, can impair immune tolerance, leading to chronic inflammation and tissue damage. In GC, IL-1 promotes immune escape by establishing an immunosuppressive microenvironment which enables tumor cells to escape immune surveillance, making immunotherapeutic approaches difficult.

IL-1 enhances the immune response in gastritis by promoting antigen presentation through the activation of dendritic cells (188) and macrophages (189). However, excessive IL-1 can damage the gastric mucosa (114). In GC, tumors manipulate IL-1 to interfere with the presentation of antigens, weaken the immune response, and facilitate immune escape (190).

In GC in particular, IL-1 is a promising target for immunotherapy. Inhibitors of IL-1 have shown the potential to reduce the immune escape of the tumor and to increase the activity of T cells (191). However, to develop effective treatments for gastritis and GC, it is critical to balance its pro-inflammatory and immunosuppressive effects.

#### 3.1.3 The future of IL-1

Going forward, targeted therapies targeting IL-1 are poised to become a key strategy in treating GC. Novel IL-1 inhibitors or combination therapies with other immunotherapies could be developed to more effectively regulate the TME and restore the anti-tumor function of the immune system by gaining a deeper understanding of the mechanisms by which IL-1 modulates the TME. Optimizing the efficacy of IL-1 inhibitors, improving their selectivity and exploring their potential synergistic effects with other immunotherapeutic agents are expected to be the focus of future research. For GC and other cancers associated with chronic inflammation, these advances may provide new therapeutic options.

### 3.2 IL-2

IL-2 plays a key role in the TME and is an important immunomodulatory factor. IL-2 maintains the immune response mainly by promoting T-cell proliferation, activation and survival, and also has a major influence on immune tolerance and immunosuppression mechanisms (192). The most important are the high affinity IL-2Rα, IL-2Rβ and IL-2Rγ (193).

#### 3.2.1 Role of IL-2 in gastritis and GC

IL-2 helps activate T cells and NK cells, leading to effective pathogen clearance in *H. pylori*-infected gastritis (194). However, excess IL-2 also promotes the expansion of regulatory T cells, which interfere with the resolution of inflammation and contribute to a pro-tumor environment (195), highlighting the dual role of IL-2 in immunomodulation.

By promoting both anti-tumor immunity and immune tolerance, IL-2 plays a key role in GC. Early on, IL-2 promotes activation of effector T and NK cells, which are essential for targeting and eliminating tumor cells (117). IL-2 also stimulates T cells to proliferate, contributing to immune tolerance and cancer progression (117). This dual role of IL-2 highlights the need for a balanced immune response to effectively fight cancer and avoid immune suppression.

#### 3.2.2 The role of IL-2 in the TME

The function of IL-2 in the TME is twofold. Especially in tumor immunotherapy, where the use of IL-2 sometimes significantly increases the therapeutic effect, IL-2 promotes the proliferation and activation of effector T cells (196) and enhances anti-tumor (197), antiviral (198) and antibacterial immune responses (199). IL-2 is also important for the expansion of regulatory T cells that maintain immune tolerance (196) and prevent autoimmune reactions by secreting immunosuppressive cytokines (192, 200) (eg, TGF-β, IL-10). Therefore, to avoid excessive immune response or immune escape, the level and role of IL-2 in the TME must be maintained at an appropriate balance.

However, immunosuppressive factors in the TME such as TGF-β and PD-L1 may block the effect of IL-2 (201). For this reason, IL-2directed immunotherapy strategies often need to be combined with other immune checkpoint inhibiting or immune-enhancing agents to optimize therapeutic efficacy. In addition, an in-depth understanding of the complex mechanisms of IL-2 action in the TME is important to improve immunotherapy, as the effects of IL-2 on the TME are also regulated by its interactions with different immune cells.

#### 3.2.3 The future of IL-2

IL-2 has a promising future in immunotherapy, particularly for cancer, autoimmune and infectious disease. Optimizing IL-2 delivery methods to enhance its anti-tumor effects while minimizing side effects through adjustments in dosage and delivery strategies will likely be the focus of future studies. In addition, by regulating T-cell function, restoring the balance of the immune system and alleviating disease symptoms, IL-2’s role in immune tolerance represents a novel approach to the treatment of autoimmune diseases. In addition, by enhancing local immune responses and improving therapeutic outcomes, IL-2 is expected to contribute to the development of vaccines and the treatment of infectious diseases. Therefore, to pave the way for more targeted and effective immunotherapy strategies, a deeper understanding of the mechanisms of IL-2 will be critical.

### 3.3 IL-4

IL-4 is a key cytokine secreted by immune cells such as Th2 cells, mast cells, and eosinophils, and it plays a crucial role in regulating the TME (202). Its primary function is to drive a Th2-type immune response by promoting B cell differentiation into plasma cells, which secrete antibodies, while simultaneously suppressing Th1-type immune responses. IL-4 also has significant roles in anti-inflammatory processes (203), fostering immune tolerance (204), and facilitating immune escape mechanisms (205).

#### 3.3.1 Role of IL-4 in gastritis and GC

Through modulation of the Th1/Th2 balance, IL-4 is a regulator of the TME in gastritis (206). In *H. pylori* infection, it promotes a Th2 response, reduces inflammatory cytokines such as IFN-γ, and limits gastric damage (118). IL-4 also supports B cell differentiation (207) and eosinophil recruitment (208). However, chronic expression of IL-4 can perpetuate inflammation, facilitate the persistence of *H. pylori*, and increase the risk of progression to GC (194).

In GC, IL-4 promotes an immunosuppressive microenvironment by polarizing M2 macrophages and promoting Treg expansion (209). This suppresses effector T and NK cell activity. IL-4 also upregulates PD-L1 in tumor cells, which impairs antigen presentation and promotes the escape of the immune system. In addition, tumor proliferation, invasion and metastasis are enhanced by IL-4-activated (210). For GC immunotherapy, targeting the IL-4 signaling pathway offers potential.

#### 3.3.2 The role of IL-4 in the TME

IL-4 secreted by Th2 cells not only promotes the activation, proliferation, and secretion of antibodies but also suppresses the cytotoxic immune response by Th1 cells (211). In allergic diseases (212), parasitic infections (213) and the TME of tumors (214), this effect is particularly pronounced.

Stimulated by IL-4, M2 macrophages secrete immunosuppressive factors to reduce inflammatory responses while supporting tissue repair by remodeling the ECM and enhancing neovascularization (215, 216). In the TME, however, M2-type macrophages can have pro-tumorigenic effects by promoting tumor cell growth, promoting immune escape, and inhibiting the immune response (209).

IL-4 affects not only immune cells but also nonimmune cells such as fibroblasts, epithelial and endothelial cells. In chronic inflammatory and fibrotic diseases, IL-4 promotes the fibrotic process through stimulation of fibroblast proliferation and collagen secretion (217, 218).

In the TME, IL-4 has a dual role to play. On the one hand, it has a pro-tumorigenic effect by promoting the escape of the immune system and supporting the proliferation of tumor cells (219). On the other hand, IL-4 can also exert an inhibitory effect on certain tumors by modulating the activity of immune cells (220). Therapeutic strategies targeting IL-4 or its pathway have potential in antitumor immunotherapy.

#### 3.3.3 The future of IL-4

As a key regulator of the immune system, the dual role of IL-4 in the regulation of inflammation and tumor immunity provides a broad perspective for future research and treatment. Further exploration of the IL-4 pathway, especially its interaction with other signal transduction networks, will help to elucidate its complex functions in the immune milieu. At the same time, new avenues for regulating inflammation and restoring anti-tumor immunity may be explored through the development of therapeutic strategies targeting IL-4 or its receptors, such as IL-4 antagonists, ADCs or small molecule inhibitors. Furthermore, combining IL-4 blockade strategies with existing immunotherapeutic approaches [e.g. immune checkpoint inhibitors (221) or CAR-T therapy (222)] may improve therapeutic efficacy and advance clinical intervention for gastritis, GC and other related diseases.

### 3.4 IL-6

IL-6 is a multifunctional inflammatory cytokine secreted by a variety of cells including macrophages, monocytes, fibroblasts and tumor cells (223). It promotes the production of acute phase proteins and the recruitment of immune cells in acute inflammation, while in chronic inflammation it can be a trigger for tissue damage and disease progression. In cancer development and progression (224), IL-6 can promote tumor cell proliferation, anti-apoptosis and angiogenesis by activating JAK/STAT3 and other signaling pathways (225, 226). At the same time, IL-6 can inhibit anti-tumor immune responses.

#### 3.4.1 Role of IL-6 in gastritis and GC

IL-6 is a pro-inflammatory cytokine that is central to the immune response to *H. pylori* infection, the most common cause of gastritis (227). It promotes the recruitment of immune cells such as macrophages and neutrophils to the gastric mucosa and contributes to the activation of inflammatory pathways (227). This exacerbates tissue damage and inflammation through the release of additional inflammatory mediators. Prolonged IL-6 signaling may lead to chronic inflammation that impairs mucosal healing and promotes progression of gastritis to pre-cancerous states such as atrophic gastritis or intestinal metaplasia (228).

In GC, IL-6 plays a dual role in tumor progression and in the modulation of the immune system. It promotes cancer growth through activation of the STAT3 pathway, enhancing cell proliferation, survival, angiogenesis and metastasis (229). In addition, IL-6 contributes to immune evasion by promoting the expansion of MDSCs (230) and regulatory T cells (231). This attenuates anti-tumor immune responses. Chronic elevation of IL-6 in the TME also maintains the inflammatory state and creates a niche that is favorable for the progression of cancer.

#### 3.4.2 The role of IL-6 in the TME

IL-6 can not only participate in inflammatory response, but also promote tumorigenesis and development in the TME. In gastritis, IL-6 mainly affects the damage and repair process of gastric mucosa by activating the JAK/STAT3 signaling pathway, regulating inflammatory response and immune cell differentiation (225). In GC, IL-6 enhances the proliferation and anti-apoptosis of tumor cells by reshaping the TME, helping them evade the clearance of the immune system (232). Therefore, IL-6 plays a crucial role in the TME of gastritis and GC.

*H. pylori* infection induces IL-6 secretion, which protects the gastric mucosa from acute inflammation, but long-term IL-6 signaling can lead to chronic inflammation and increase the risk of GC (233). In GC, IL-6 promotes the activation of TAMs and CAFs, which further enhance the inflammatory response by secreting IL-6 and other factors, creating a vicious cycle (227, 234). In addition, IL-6 directly promotes the proliferation, survival, and invasion of tumor cells by activating STAT3 signaling (223).

IL-6 impairs immune surveillance of tumors by inducing T cells differentiation and inhibiting the activity of effector T cells (235). In addition, IL-6 can also inhibit the maturation and antigen presentation function of dendritic cells, further reducing the immune system’s ability to respond to pathogens or tumor cells (223). High levels of IL-6 in chronic gastritis may lead to the immune system’s tolerance to *H. pylori*, creating the conditions for the persistence of inflammation and the development of GC. In addition, IL-6 can help tumor cells achieve immune escape through a variety of pathways (236).

In conclusion, IL-6 has an important dual role in the TME of gastritis and GC.

#### 3.4.3 The future of IL-6

Although IL-6 has a role in fighting inflammation and supporting immune defense, its tumori-promoting effect in GC makes it an important target for immunotherapy. In the future, it is expected that the treatment strategies for gastritis and GC will be optimized by precisely regulating the IL-6 signaling pathway, combined with immune checkpoint inhibitors or other treatments, and providing patients with more effective clinical interventions.

### 3.5 IL-10

IL-10 is an anti-inflammatory cytokine that is mainly secreted by regulatory T cells, B cells, monocytes, and TAMs, and plays an important role in maintaining immune homeostasis and inhibiting excessive inflammation (237).

#### 3.5.1 Role of IL-10 in gastritis and GC

In the early stage of *H. pylori*-induced gastritis or gastritis caused by other stimuli, immune cells such as macrophages and Th1 cells release large amounts of pro-inflammatory factors, including TNF-α, IL-1β, and IFN-γ. IL-10 downregulates the expression of these factors by activating the STAT3 pathway. This effectively alleviates the mucosal inflammatory response and reduces tissue damage. Meanwhile, IL-10 inhibits the antigen-presenting function of DCs and macrophages. It also reduces CD4+ T cell activation and decreases chemokine expression. Thus, IL-10 controls the excessive infiltration of immune cells into the gastric mucosa and prevents the spread of inflammatory responses (118, 124). However, persistent expression of IL-10 allows *H. pylori* to evade the immune system, maintain infection and create a microenvironment conducive to GC progression (238). Elevated levels of IL-10 may reduce bacterial immune clearance and increase cancer risk in chronic *H. pylori* gastritis. IL-10 from B cells has been associated with an accelerated rate of progression of GC.

#### 3.5.2 The role of IL-10 in the TME

Within the complex milieu of the TME in cancer, IL-10 can exhibit a dichotomous role, exhibiting antagonistic and stimulatory properties in distinct contexts. Specifically, IL-10 has been shown to reduce chronic inflammation, thereby lowering the risk of tumorigenesis. Conversely, elevated levels of IL-10 within the TME can impede effective anti-tumoral immune responses, thus facilitating immune evasion and tumor progression (239).

#### 3.5.3 The future of IL-10

Due to its potent anti-inflammatory properties, IL-10 holds great promise for therapeutic applications in inflammation, cancer and autoimmune diseases. Strategies are being developed to improve the stability and delivery of IL-10 derivatives to effectively modulate the immune balance in autoimmune diseases such as rheumatoid arthritis (240) and inflammatory bowel disease (241). In cancer, IL-10’s dual role is being intensively studied, particularly its potential to enhance antitumor responses with immune checkpoint inhibitors. Targeting IL-10 therapeutics to improve efficacy and minimize side effects is possible through advances in (242) and precision delivery systems (243). Personalized therapies for immune-related diseases may emerge from further research into the signaling pathways and regulatory mechanisms of IL-10.

### 3.6 IL-12

IL-12 is a key pro-inflammatory cytokine that regulates immune responses and is secreted by antigen-presenting cells such as dendritic cells and macrophages (244). It promotes the differentiation of CD4+ T cells into Th1 cells (245). It drives the production of IFN-γ and enhances cell-mediated immunity (246). In addition, bridging innate and adaptive immunity, IL-12 activates NK cells and enhances their cytotoxic and antitumor functions (247). In the TME, IL-12 inhibits tumoral growth and supports anti-tumoral immunity. However, underscoring the need for balanced IL-12 expression, excessive IL-12 can lead to harmful inflammation and has been linked to autoimmune diseases (248).

#### 3.6.1 Role of IL-12 in gastritis and GC

It has been established that IL-12 plays a crucial role in the immune response associated with gastritis, particularly in cases of *H. pylori* -induced gastritis. As a pro-inflammatory cytokine, IL-12 facilitates the differentiation of CD4+ T cells into Th1 cells, thereby enhancing the production of IFN-γ, which, in turn, accelerates the eradication of *H. pylori* (249). However, the predominance of this Th1-type immune response can also intensify gastric inflammation, thereby contributing to mucosal damage (250). The persistent inflammation that is driven by IL-12 has been demonstrated to heighten the risk of progression from gastritis to gastric atrophy, and eventually, GC, thereby underscoring its dualistic role in both protecting against infection and contributing to disease progression.

#### 3.6.2 The role of IL-12 in the TME

Within the TME, IL-12 has been shown to regulate immune cell function, activate effector T and NK cells, and augment anti-tumor immune responses. By inducing a Th1-type immune response, IL-12 contributes to enhancing cell-mediated immune responses and impeding the growth and metastasis of tumor cells (251). Furthermore, IL-12 has been observed to enhance antigen presentation via its modulation of dendritic cells (252), thereby contributing to the initiation and sustenance of immune surveillance within tumors. Nevertheless, immunosuppressive factors in the TME have the potential to impede the effects of IL-12 and curtail its therapeutic potential (253).

Notwithstanding the capacity of IL-12 to augment the immune response, tumor cells have the capacity to inhibit the action of IL-12 through a variety of mechanisms, thereby leading to immune evasion. Immunosuppressive cells within the TME, such as regulatory T cells (254) and M2 macrophages (255), may hinder the pro-inflammatory effects of IL-12 by secreting cytokines like IL-10 (256), thereby diminishing the strength of the immune response. Furthermore, prolonged IL-12 activation has been shown to induce immune tolerance, a process that can impede the immune system’s capacity to recognize and combat tumor cells, thus creating a favorable environment for tumor cell proliferation and immune evasion (257).

#### 3.6.3 The future of IL-12

It is reasonable to hypothesize that in the future, immunotherapy strategies that target IL-12 will become more sophisticated. Research is anticipated to prioritize optimizing targeted delivery of IL-12 through genetic engineering, reducing systemic adverse effects, and enhancing its efficacy in the TME. A promising avenue for advancement in GC and other tumors may lie in the combination of IL-12 with other immunotherapy methods, such as immune checkpoint inhibitors (258) and CAR-T cell therapy (259). The significance of IL-12 in the realm of tumor immunotherapy is anticipated to be further underscored by advancements in precision medicine and targeted delivery methodologies.

### 3.7 IL-17

The IL-17 class of pro-inflammatory cytokines is secreted by Th17 cells and their derivatives, including gamma delta T cells and natural killer T cells (260). These cytokines play a pivotal role in regulating inflammatory responses. The IL-17 family comprises IL-17A, IL-17B, IL-17C, IL-17D, IL-17E, and IL-17F (260). Among them, IL-17A is regarded as the most representative and the most extensively studied member. By binding to its receptor, designated as IL-17R, IL-17 triggers the activation of multiple signaling pathways, resulting in the promotion of downstream cytokine production and leukocyte recruitment. This phenomenon manifests a dual effect on both immune response and tissue damage (261).

#### 3.7.1 Role of IL-17 in gastritis and GC

By promoting an inflammatory response that recruits and activates immune cells such as neutrophils and macrophages, IL-17 plays a central role in *H. pylori*-induced gastritis. IL-17 is critical for the elimination of *H. pylori* (156). However, its overactivity can lead to chronic inflammation, creating an environment conducive to the development of GC. Particularly in individuals with gastritis, elevated levels of IL-17 correlate with an increased risk of GC. IL-17 plays a dual function in the development of gastritis and cancer: in the early stages, IL-17 can contribute to tumor cell killing, but in the tumor environment, IL-17 supports immune evasion and promotes tumor cell survival and growth through modulation of immune cell function (262, 263).

Studies have shown that by promoting inflammatory responses, activating immune cells and inducing the release of pro-inflammatory factors, IL-17 is able to drive GC development and progression (158). The dual role of IL-17 in GC makes it a potential target for research and therapy.

#### 3.7.2 The role of IL-17 in the TME

IL-17, produced by Th17 cells, γδ T cells and other immune cells, is central to inflammation, immunity and tissue repair through binding to its receptor, IL-17R, and activation of downstream pathways. It enhances local immune defense against pathogens by inducing the secretion of pro-inflammatory cytokines. Sustained IL-17 activity may drive chronic inflammation (264) and contribute to cancer (156), autoimmunity (265) and fibrotic disorders (266). In addition, IL-17 regulates immune cell interactions by influencing the balance of Th17 and Treg and promoting immune suppression via MDSCs (267). This facilitates immune escape in tumors.

By stimulating fibroblasts, collagen synthesis and ECM remodeling, IL-17 also supports tissue repair (268). These processes can exacerbate pathological fibrosis and tissue damage in chronic conditions such as cancer and fibrosis (266). While the role of IL-17 is protective, its dysregulation poses challenges. Therapeutic approaches that target the IL-17 pathway are promising but require careful management to balance benefits and risks.

#### 3.7.3 The future of IL-17

Hitherto, research on IL-17 has focused on its role in immune modulation. By leveraging an enhanced comprehension of the IL-17 signaling pathway, the development of more precise treatment methodologies can be facilitated. These methodologies hold promise in reducing adverse effects and enhancing the precision of treatment, thus improving patient outcomes. Moreover, the potential synergistic effect of IL-17 when employed in conjunction with other immunotherapy modalities, such as with immune checkpoint inhibitors (269), warrants further exploration. Consequently, the therapeutic potential of IL-17 in tumor immunotherapy merits further investigation, as it could offer novel concepts and strategies for the management of GC, among other types of tumors.

### 3.8 IL-23

IL-23 is a pro-inflammatory cytokine that plays a pivotal role in the TME, primarily through the regulation of Th17 cell differentiation and function (270). Its function includes the maintenance of Th17 cell expansion through the activation of the JAK-STAT pathway, the promotion of inflammatory factor production (e.g., IL-17 and IL-22), and, consequently, the enhancement of mucosal barrier defense and pathogen clearance (271). However, uncontrolled activation of IL-23 has been associated with the pathogenesis of various autoinflammatory conditions, including psoriasis (272) and inflammatory bowel disease (248). Within the TME, IL-23 exhibits a dual role, functioning both to enhance anti-tumor immunity and to promote tumor progression through the mechanisms of chronic inflammation and immune escape (273). Consequently, IL-23 represents a significant target for the therapeutic management of inflammatory diseases and demonstrates potential value in the context of tumor immunotherapy.

#### 3.8.1 Role of IL-23 in gastritis and GC

In *H. pylori* -induced gastritis, IL-23 drives chronic inflammation by promoting the differentiation of Th17 cells, which in turn produce pro-inflammatory cytokines such as IL-17 (263). This cytokine cascade damages the gastric mucosa and impedes healing. This contributes to chronic gastritis. Persistent IL-23 activation is a potential target for therapeutic intervention because it exacerbates inflammation and may perpetuate *H. pylori* infection.

In GC, IL-23 has a dual role. Through Th17-mediated tumor surveillance, it can enhance antitumor immunity. Chronic IL-23 activation promotes a proinflammatory milieu that is conducive to angiogenesis (274), and cancer progression (170). The complex role of IL-23 in GC is underscored by the interplay between its protective and tumor-promoting effects.

#### 3.8.2 The role of IL-23 in the TME

In the TME, IL-23 is a key player in chronic inflammatory conditions and autoimmune diseases. It maintains the inflammatory milieu and immune cell activation. IL-23 has been shown to cause tissue damage and chronic inflammation, making people more prone to cancer.

In the context of cancer, the role of IL-23 is more complex. On the one hand, by activating Th17 cells and NK cells that can recognize and kill cancer cells, it can enhance the immune system’s ability to fight tumors (247, 275). On the other hand, persistent IL-23 activity can contribute to a chronic inflammatory environment that is conducive to tumor growth and progression through the promotion of angiogenesis (276) and immune evasion (277). Thus, depending on the specific context and balance of immune responses, IL-23 is a double-edged sword in the TME.

#### 3.8.3 The future of IL-23

Particularly in the treatment of autoimmune diseases, chronic inflammation and cancer, the future of IL-23 research holds significant therapeutic potential. Given its critical role in driving Th17 cell differentiation and perpetuating inflammation, IL-23 is a target for therapeutic intervention in diseases like psoriasis. In clinical trials, monoclonal antibodies that inhibit IL-23 signaling have shown promise. In cancer, the pro-inflammatory effects of IL-23 can also promote tumor growth, although IL-23 may stimulate anti-tumor immunity. The refinement of IL-23 modulation strategies to exploit its therapeutic benefits while minimizing its potential to promote chronic inflammation or immune evasion in cancer will likely be the focus of future research.

## 4 TNF

TNF is a master regulator of inflammatory responses, produced primarily by macrophages, dendritic cells and T cells (278). TNF binds to TNFR1 and TNFR2 to mediate its effects (279). It plays a critical role in acute inflammation by promoting the activation of the endothelium and the adhesion and migration of leukocytes to the sites of inflammation (280). TNF stimulates the production of pro-inflammatory cytokines, and thus amplifies the immune response. However, excessive or chronic TNF activity is characteristic in autoimmune diseases, such as rheumatoid arthritis (281) and inflammatory bowel disease (282), where it drives tissue damage and systemic inflammation. The specific mechanism of TNF in GC and gastritis is detailed in **Table 3**.

**Table 3:** The mechanism of TNF in gastritis and GC.

Cytokines	Brief biological mechanism in gastritis	Brief biological mechanism in GC
TNF-α	*H. pylori* infection promotes M1 macrophage polarization and gastric inflammation by activation of NLRP3 inflammasome via TNF/TNFR1 axis (283) Exopolysaccharide54 could effectively alleviate the gastritis in the *H. pylori*-infected mice by down-regulating the mRNA expression levels of TNF-α in gastric cell (284) TNF-alpha is involved in pathogenesis of gastritis induced by Helicobacter felis infection as IFN-gamma (285)	Tipα secreted from *H. pylori* stimulates GC development by inducing TNF-α, an endogenous tumor promoter, through its interaction with nucleolin, a Tipα receptor (286) TNFα might activate TLR2-β-catenin-signaling in GC (287) The TNF-α/TNFR2 pathway increases the expression of Foxp3 and the production of TGF-β in T cells in the GC microenvironment (288) Oridonin suppresses GC SGC-7901 cell proliferation by targeting the TNF-alpha/androgen receptor/TGF-beta signalling pathway axis (289)

### 4.1 Role of TNF in gastritis and GC

TNF drives inflammation in *H. pylori* -induced gastritis by activating the NF-κB pathway, stimulating the release of other inflammatory cytokines (288, 290). This results in infiltration of immune cells and damage to the stomach lining. Chronic elevated TNF contributes to persistent inflammation and compromises mucosal repair, laying the foundation for GC (278).

In GC, TNF drives tumor progression through NF-κB and MAPK pathways, promotes angiogenesis, cell proliferation and metastasis, and suppresses antitumor immunity (291). Reflecting its dual function, despite its pro-tumor role, TNF also has apoptotic effects on tumor cells (278).

### 4.2 The role of TNF in the TME

In the TME of a tumor, TNF-α plays a complex dual role, both as an inhibitor of tumorigenesis and, under certain conditions, as a potential promoter of tumor progression.

By activating cytotoxic T cells and NK cells, TNF-α enhances its anti-tumor effects. In addition, TNF-α induces tumor cell expression of death receptors (e.g., Fas (292) and TNFR1 (293)), which initiates apoptosis through extracellular pathways and inhibits tumor growth. On the one hand, TNF-α plays a key role in enhancing the antigen-presenting function and promoting the release of inflammatory factors, thereby providing the body with an effective anti-tumor immune environment (294).

Under conditions of chronic inflammation, TNF-α supports tumor development and proliferation through multiple mechanisms. First, TNF-α is able to promote angiogenesis and tumor invasion through the up-regulation of VEGF (295) and MMPs (296). Second, TNF-α suppresses the activity of effector T cells by recruiting immunosuppressive cells such as regulatory T cells (235) and MDSCs (297), creating an immune escape environment. In addition, TNF-α activates the M2-type polarization of TAMs (298) and secretes inhibitory factors such as IL-10 and TGF-β, further suppressing anti-tumor immune responses.

In summary, depending on its concentration, local environment and regulatory status of signaling pathways, the role of TNF-α in the TME varies. Therapeutic strategies based on TNF-α need to enhance its anti-tumor ability while at the same time avoiding its tumor-promoting effects. In recent years, new ideas for optimizing tumor immunotherapy have emerged, such as combination therapy targeting TNF-α signaling (299).

### 4.3 The future of TNF

The future of TNF research is aimed at optimizing its therapeutic potential, particularly in autoimmune diseases and cancer treatment. Efforts are focused on refining TNF-targeted therapies to minimize side effects and improve outcomes. In cancer, the combination of TNF modulation with immune checkpoint inhibitors is being explored to boost anti-tumor immunity while addressing its role in chronic inflammation and immune tolerance. Understanding the dual role of TNF in disease progression is essential for the development of more effective, targeted therapies.

## 5 IFN

IFNs are a family of cytokines that play a key role in the regulation of the immune system and are divided into three types: Type I (e.g., IFN-α, IFN-β), Type II (IFN-γ), and Type III (IFN-λ) (300). In response to infection, stress and malignancy, these cytokines are produced (301, 302). Their primary role in inflammation is to initiate an antiviral response, to modulate the function of immune cells, and to influence the inflammatory cascade. The specific mechanism of IFNs in GC and gastritis is detailed in **Table 4**.

**Table 4:** The mechanism of IFN in gastritis and GC.

Cytokines	Brief biological mechanism in gastritis	Brief biological mechanism in GC
IFN-α	IFN-α inhibits gastric acid secretion centrally through nitric oxide pathways probably mediated (303)	IFN-α sensitizes human GC cells to TRAIL-induced apoptosis via activation of the c-CBL-dependent MAPK/ERK pathway (304) IFN-α enhanced 5’-DFUR-induced apoptosis in GC cells by upregulation of TP expression, which is partially regulated by activation of ERK signaling (305)
IFN-β	―	Cytosine deaminase and IFN-β genes in the presence of 5-fluorocytosine have significant synergistic anticancer effects (306)
IFN-γ	IFN-γ as a critical promoter of parietal cell atrophy with metaplasia during the progression of gastritis to gastric atrophy and metaplasia (307) Gastric infection and inflammation are associated with increased IFN-γ expression and reduced ghrelin expression (308)	sLAG-3 might inhibit the tumor growth, and promote the secretion of CD8+T cells, IL-12 and IFN-γ (309) A combination of cyclosporin-A and IFN-γ induces apoptosis in human gastric carcinoma cells (310) IFN-gamma suppressed cell growth through induction of both cell cycle arrest and apoptosis (311)

### 5.1 Role of IFN in gastritis and GC

In gastritis caused by *H. pylori*, IFNs play an important role in the immune response. The inflammatory response induces the production of these cytokines, which increase local inflammation and recruit other immune cells (T cells, NK cells) through macrophage/dendritic cell activation (312). In *H. pylori*-induced gastritis, the expression of IFN-γ is elevated, enhancing the antimicrobial immune response. IFN-γ induces the expression of PD-L1, which contributes to limiting persistent inflammation and alleviating gastric mucosal tissue damage. However, PD-L1 binds to PD-1 on T cells, leading to T cell exhaustion and suppression of the immune response. This ultimately results in an immunosuppressive microenvironment that promotes tumor cell survival, metastasis, and therapeutic resistance (313). Recombinant forms of IFN-α have been used to treat melanoma (314), renal cell carcinoma (315), and GC (316) because of their ability to induce tumor cell apoptosis and enhance immune activation. However, chronic IFN signaling in the GC microenvironment may enhance tumor progression by promoting vascularization and tumor survival via pathways including VEGF and TGF-β (317, 318).

### 5.2 The role of IFN in the TME

IFN activate immune responses by modulating the activity of immune cells and influencing the TME. Type I IFNs (IFN-α/β) activate antigen presentation, enhance NK cell and macrophage function, and stimulate the expression of ISGs to establish an antiviral state. They play an essential role in early immune responses to infections and tumors (319). Type II IFN (IFN-γ), produced mainly by T and NK cells, promotes Th1 differentiation, macrophage activation and antigen presentation, which are critical for controlling infection and tumor growth (320). However, excessive or prolonged IFN signaling can induce chronic inflammation, tissue damage and immune dysregulation (321). In the TME, prolonged IFN exposure can upregulate immune checkpoint molecules such as PD-L1, leading to immune tolerance and facilitating immune escape (322). In addition, prolonged IFN signaling may promote tumor cell survival, angiogenesis, and metastasis, complicating its therapeutic use. The balance between immune activation and suppression driven by IFNs is critical in cancer and chronic inflammatory diseases.

### 5.3 The future of IFN

Improving their therapeutic applications, particularly in cancer, viral infections and autoimmune diseases, is the future of IFNs in medical research. New approaches aim to refine the use of IFNs to enhance immune responses against tumors. Combinations of IFNs and immune checkpoint inhibitors show promise in boosting anti-tumor immunity. Researchers are also investigating strategies to minimize the adverse effects of prolonged IFN signaling, which can contribute to chronic inflammation and immune tolerance. Future therapies may offer more effective and targeted solutions for a variety of immune-related diseases through a better understanding of the mechanisms of IFN signaling in the TME and autoimmune contexts.

## 6 Chemokines

Chemokines are a class of small signaling proteins that play important roles in the immune response, primarily by directing immune cell migration to sites of infection, inflammation, or injury (323). They play a critical role in immune surveillance (324), tissue homeostasis (325), and development of the immune system (326) and receptor signaling in cancer (327). The specific mechanism of Chemokines in GC and gastritis is detailed in **Table 5**.

**Table 5:** The mechanism of chemokines in gastritis and GC.

Cytokines	Brief biological mechanism in gastritis	Brief biological mechanism in GC
CCL2	*H. pylori* induce eosinophil migration through the chemokine CCL2, which in turn causes gastritis (328)	PDPN+ cancer-associated fibroblasts enhance GC angiogenesis via the CCL2-ACKR1 axis (329) Ephrin A1 Stimulates CCL2 Secretion to Facilitate Pre-metastatic Niche Formation and Promote GC Liver Metastasis (330) CCL2 expression correlates closely with HIF-1α expression in GC (331)
CCL3	*H. pylori* infection stimulates macrophages to secrete CCL3 through the JAK1-STAT1 pathway and disrupts gastric epithelial tight junctions through phosphorylation of P38, resulting in gastritis (332)	CCL3 and CCL20-recruited dendritic cells modified by melanoma antigen gene-1 induce anti-tumor immunity against GC (333)
CCL5	CCL5(+) T cells, presumably activated cytotoxic T cells, would play important roles in the active inflammatory process of chronic gastritis (334)	A novel long noncoding RNA, TMEM92-AS1, promotes GC progression by binding to YBX1 to mediate CCL5 (335) Down-regulation of KLF5 in cancer-associated fibroblasts inhibit GC cells progression by CCL5/CCR5 axis (336) 17β-estradiol inhibits mesenchymal stem cells-induced human AGS GC cell mobility via suppression of CCL5- Src/Cas/Paxillin signaling pathway (337)
CXCL8	Streptococcus anginosus is a gram-positive coccus that leads to the upregulation of the pro-inflammatory chemokine CCL8, which has long-term effects on gastric barrier function and microbiota homeostasis, resulting in superficial gastritis (338)	Guanylate binding protein 5 accelerates GC progression via the JAK1-STAT1/GBP5/CXCL8 positive feedback loop (339)
CXCL10	Palmatine ameliorates N-methyl-N’-nitrosoguanidine-induced chronic atrophic gastritis through the STAT1/CXCL10 axis (340)	Huang-Jin-Shuang-Shen Decoction promotes CD8+ T-cell-mediated anti-tumor immunity by regulating chemokine CXCL10 in GC (341) CXCL10 and IL15 co-expressing chimeric antigen receptor T cells enhance anti-tumor effects in GC by increasing cytotoxic effector cell accumulation and survival (342) Targeting Autophagy Facilitates T Lymphocyte Migration by Inducing the Expression of CXCL10 in GC Cell Lines (343) CXCL10/CXCR3 axis promotes the invasion of GC via PI3K/AKT pathway-dependent MMPs production (344)
CXCL12	Upexpression of BHLHE40 in gastric epithelial cells increases CXCL12 production through interaction with p-STAT3 in *H. pylori* -associated gastritis (345)	The circular RNA circDLG1 promotes GC progression and anti-PD-1 resistance through the regulation of CXCL12 by sponging miR-141-3p (346) Cancer-associated fibroblasts in GC affect malignant progression via the CXCL12-CXCR4 axis (347) miR-1273h-5p suppresses CXCL12 expression and inhibits GC cell invasion and metastasis (348) MicroRNA-200b-3p restrains GC cell proliferation, migration, and invasion via C-X-C motif chemokine ligand 12/CXC chemokine receptor 7 axis (349)
CX3CL1	Cytotoxin-associated gene A-Negative *H. pylori* promotes gastric Mucosal CX3CR1CD4 Effector Memory T Cell recruitment in mice, causing gastritis (350)	Lactate/GPR81 recruits regulatory T cells by modulating CX3CL1 to promote immune resistance in a highly glycolytic GC (351) Overexpression of CX3CR1 is associated with cellular metastasis, proliferation and survival in GC (352)

### 6.1 CCL2

CCL2 also known as MCP-1, is an important chemokine (353). It is a member of the C-C motif chemokine family. It promotes the chemotaxis of immune cells, in particular monocytes, macrophages and dendritic cells, by binding to its receptor CCR2.CCL2 (354) plays an important role in a wide variety of physiological and pathological processes, including inflammation, the immune response, the TME and immune escape (354).

#### 6.1.1 Role of CCL2 in gastritis and GC

In gastritis, especially chronic gastritis caused by *H. pylori*, the role of CCL2 is particularly prominent. Infection with *H. pylori* stimulates the gastric mucosa to produce CCL2, which in turn attracts monocytes and macrophages to the site of inflammation (355, 356). Macrophages promote gastric mucosal injury and repair by secreting inflammatory factors such as IL-8 and JAK, which enhance the local immune response. Although CCL2 contributes to the antimicrobial immune response, its overexpression can also lead to chronic inflammation and immune dysregulation (135, 357). This may increase the risk of precancerous lesions such as GC. CCL2 plays a role in promoting the recruitment of immune cells, particularly monocytes and macrophages, in the TME of GC (358). By secreting CCL2, tumor cells induce immune cells into the TME. These immune cells, particularly TAMs, can promote tumor growth and metastasis by secreting various cytokines (e.g., IL-10, TGF-β, etc.) to maintain an immunosuppressive status in the TME (358). Macrophages not only play a role in immune escape from tumors, but also exacerbate tumor progression through promotion of angiogenesis and suppression of effector T cell function (359). Therefore, the role of CCL2 in GC may be both to initiate the immune response and to be part of the immune escape mechanism of tumors.

#### 6.1.2 The role of CCL2 in the TME

By promoting the recruitment of immunosuppressive immune cells such as TAMs and Treg cells, CCL2 contributes to tumor immune escape. Tumor cells and CAFs recruit macrophages into the TME by secreting CCL2, and these macrophages are usually M2type with immunosuppressive function (354).

Researchers are exploring immunotherapeutic strategies that target the CCL2/CCR2 pathway because of the important role of CCL2 in immune escape (360). Inhibition of the binding of CCL2 to CCR2 or blocking the production of CCL2 may decrease the accumulation of immunosuppressive cells, such as M2 macrophages, in the TME and increase effector T-cell clearance (361, 362). This targeted therapy may represent a new direction for immunotherapy of tumors such as GC, as it has shown good results in preclinical studies in several tumor types.

#### 6.1.3 The future of CCL2

In order to reduce the immunosuppressive effects in the TME and enhance the anti-tumor immune response, future studies may focus on fine-tuning the CCL2/CCR2 pathway. Furthermore, combining CCL2 with other immunotherapeutic strategies (e.g. immune checkpoint inhibitors, CAR-T cell therapies, etc.) can significantly improve immunotherapy efficacy (363). New opportunities for the treatment of GC and other tumors will be opened by optimizing the targeting of CCL2 and better understanding its complex role in the TME.

### 6.2 CCL3

CCL3 also known as MIP-1α, is an important chemokine belonging to the C-C motif chemokine family. CCL3 plays an important role in inflammation, immunomodulation, infectious diseases, and tumors (364).

#### 6.2.1 Role of CCL3 in gastritis and GC

*H. pylori* infection was found to activate immune cells in the gastric lining, leading to the production of CCL3, which promotes a local immune response by binding to CCR-1 and CCR-5 receptors and recruits immune cells including monocytes, macrophages and T cells to the site of inflammation (332). However, prolonged overexpression of CCL3 can lead to chronic inflammation, providing a permissive environment for precancerous lesions such as GC to develop (365).

CCL3 plays an important role in the TME of GC. Tumor cells recruit immune cells, particularly macrophages and T cells, into the TME through the secretion of CCL3 (333, 366). However, tumor cells can suppress anti-tumor immune responses by altering the function of immune cells. In addition, CCL3 has a role in the promotion of angiogenesis, which may increase the supply of oxygen and nutrients to tumors and promote tumor growth and metastasis (367).

#### 6.2.2 The role of CCL3 in the TME

By recruiting immunosuppressive cells such as M2-type macrophages and Treg cells, CCL3 can promote immune evasion during tumor immune escape (368). Although CCL3 can enhance local immune evasion, its recruitment of these suppressive cells can diminish effector T cell function and impair tumor cell recognition and clearance, thereby promoting tumor growth and metastasis (369). As a result, the role of CCL3 in the TME can be both supportive of the immune response and contribute to immune escape through immunosuppressive mechanisms. Targeting CCL3 with immunotherapeutic strategies, such as blocking the CCL3/CCR1/CCR5 interaction, could reduce the accumulation of immunosuppressive cells and enhance anti-tumor immune responses (369, 370). The CCL3/CCR5 pathway is a promising target for overcoming immune escape in GC, as studies suggest that CCR5 antagonists *may improve the efficacy of immunotherapy in various cancers.*

#### 6.2.3 The future of CCL3

Future studies targeting the CCL3/CCR5 signaling pathway may aim to enhance immunotherapy efficacy, particularly when combined with immune checkpoint inhibitors or CAR-T cell therapy (371, 372). Inhibiting CCL3 activity or its receptor could reduce immunosuppressive effects in the TME, restoring anti-tumor immune responses. Additionally, precise regulation of CCL3 expression in the TME may offer new therapeutic strategies for immunotherapy in GC and other malignancies.

### 6.3 CCL5

CCL5 also known as RANTES, is an important chemokine that belongs to the C-C motif chemokine family (373). It is a chemokine secreted mainly by T cells, macrophages, dendritic cells, endothelial cells and tumor cells (374). It regulates the migration of immune cells, especially immune cells such as T cells, macrophages and eosinophils, by binding to CCR1, CCR3 and CCR5 receptors (375–377). CCL5 not only promotes the aggregation of immune cells, but also enhances cell-cell interactions, thereby strengthening the immune response. In addition, CCL5 is involved in the regulation of immune cell activation, proliferation, differentiation and cytokine secretion (378).

#### 6.3.1 Role of CCL5 in gastritis and GC

The expression of CCL5 is normally increased when the gastric mucosa is infected or injured, which recruits immune cells such as T cells and macrophages to the site of inflammation and enhances the immune response (379). However, excessive CCL5 activity can lead to a persistent activation of the immune response, which can induce chronic inflammation and increase the damage to the gastric mucosa, thus providing favorable conditions for pre-cancerous lesions such as GC (379). In the TME of GC, CCL5 plays a complex dual role. On the one hand, CCL5 enhances the anti-tumor immune response by promoting the recruitment of T cells and NK cells. Studies show that high CCL5 expression has been linked to stronger anti-tumor immune responses, particularly effector T-cell and NK cell recruitment (380–382). On the other hand, by binding to the CCR5 receptor, CCL5 can recruit immunosuppressive cells such as TAMs and inhibit the function of tumor-specific T cells, thereby exacerbating tumor immune escape (336, 379). In addition, CCL5 may also support tumor growth through the promotion of angiogenesis and the enhancement of tumor cell migration and metastasis (383).

#### 6.3.2 The role of CCL5 in the TME

CCL5 can promote the immune response against tumors through the recruitment of effector cells such as T cells and NK cells, but in some TMEs it can also promote immune escape through the recruitment of immunosuppressive cells such as M2 macrophages and Treg cells (384). CCL5 recruits M2 macrophages via the CCR5 receptor. M2 macrophages secrete anti-inflammatory factors (e.g., IL-10, TGF-β) that suppress tumor-specific immunity and promote tumor survival and metastasis (385). Because of its role in immune escape, targeting the CCL5/CCR5 pathway has become a focus of immunotherapy research.

#### 6.3.3 The future of CCL5

The accumulation of suppressive cells can be reduced and anti-tumor immune responses can be enhanced by inhibiting CCL5/CCR5 binding (379). Studies have shown that CCR5 antagonists, especially when combined with immune checkpoint inhibitors or CAR-T cell therapy, can improve immunotherapy outcomes in various cancers, making the CCL5/CCR5 pathway a promising strategy for the treatment of GC (386).

### 6.4 CXCL8

CXCL8 is an important chemokine belonging to the C-X-C motif chemokine family, also known as IL-8 (387). It is predominantly secreted by various cell types including neutrophils, macrophages, endothelial cells, fibroblasts, tumor cells and others (388). By binding to its receptors CXCR1 and CXCR2, CXCL8 exerts chemotactic effects on immune cells, in particular neutrophil recruitment and activation (387). Furthermore, CXCL8 plays important roles in physiological and pathological processes including inflammation, immune response and TME (387).

#### 6.4.1 Role of CXCL8 in gastritis and GC

In the gastric mucosa, CXCL8 enhances local immune responses by promoting neutrophil chemotaxis and activation, thereby contributing to the resolution of infection (389). However, prolonged high expression of CXCL8 and excessive neutrophil recruitment can lead to chronic inflammation and damage to the gastric mucosal lining, creating conditions conducive to the development of diseases like GC (390). In addition to enhancing local inflammatory responses in the tumor by recruiting immune cells, CXCL8 may also promote tumor development by promoting tumor cell growth, angiogenesis and metastasis (391). CXCL8 recruitment and activation of neutrophils by binding to CXCR1 and CXCR2 has been shown to enhance tumor growth and proliferation through secretion of a variety of cytokines and angiogenic factor release (392, 393). In addition, by inducing the accumulation of TAMs, CXCL8 may promote immune escape from the TME (394).

#### 6.4.2 The role of CXCL8 in the TME

By regulating the migration and function of immune cells in the TME, CXCL8 may support immune escape of tumor cells (394). Targeting CXCL8 or its receptors (CXCR1 and CXCR2) has emerged as a potential immunotherapeutic strategy due to the important role of CXCL8 in immune escape. By inhibiting the binding of CXCL8 and CXCR1/2, the aggregation of immunosuppressive cells (e.g., neutrophils, TAMs, etc.) in the TME can be reduced, thereby promoting the anti-tumor activity of effector T cells (392, 393). Studies have shown that inhibition of the CXCL8 pathway has the potential to enhance the effectiveness of immunotherapy, especially when combined with immune checkpoint inhibitors or other immunotherapy (395).

#### 6.4.3 The future of CXCL8

Therapeutic strategies that precisely target the CXCL8 receptor to reduce immunosuppression in the TME and restore anti-tumor immune responses are likely to be the focus of future CXCL8 research. New ideas and therapeutic approaches for the treatment of GC and other malignancies may be provided by optimizing the role of CXCL8 in the TME.

### 6.5 CXCL12

CXCL12 also known as stromal cell-derived factor 1α, is an important chemokine (396). It belongs to the C-X-C motif chemokine family. CXCL12 can be secreted by various cell types including fibroblasts, endothelial cells, macrophages, and tumor cells (397). CXCL12 binds to the CXCR4 and CXCR7 receptors and is involved in many physiological and pathologic processes, including immune response, cell migration and tumor metastasis (397, 398).

#### 6.5.1 Role of CXCL12 in gastritis and GC

When infected by *H. pylori*, the stomach produces CXCL12, which recruits immune cells such as T cells and macrophages to the inflamed area (399). CXCL12 helps resolve the infection by regulating immune cell localization and activation through binding to CXCR4 and CXCR7 receptors (400). However, excessive expression of CXCL12 can lead to chronic inflammation, which can damage the lining of the stomach and increase the risk of pre-cancerous lesions such as GC (401). In the TME of GC, CXCL12 plays a dual role. First, by recruiting immune cells to the TME, CXCL12 enhances the immune response (347). In some cases, CXCL12 expression may enhance effector T cells, NK cells, and other antitumor immune function (402). However, CXCL12 can also promote tumor metastasis by facilitating the migration and invasion of tumor cells. Tumor cells, CAFs, and others may secrete CXCL12 and activate the CXCR4 receptor, which directs tumor cells to specific sites and promotes metastatic and neovascular growth (403, 404).

#### 6.5.2 The role of CXCL12 in the TME

By recruiting immunosuppressive cells such as Treg cells and M2 macrophages, the CXCL12/CXCR4 signaling pathway plays a critical role in tumor immune escape (400). High CXCL12 expression has been implicated in immune escape, metastasis and drug resistance in several tumor types, including GC (405). CXCL12 promotes immunosuppression by recruiting CAFs and reducing effector T-cell and NK-cell function (406).

#### 6.5.3 The future of CXCL12

Targeting the CXCL12/CXCR4 signaling pathway by inhibiting their binding or by blocking the expression of CXCL12 can reduce the accumulation of immunosuppressive cells and enhance the anti-tumor immunity. This pathway is a promising therapeutic target as studies have shown that CXCR4 antagonists can improve immune responses and slow tumor progression.

### 6.6 CXCL10

CXCL10, also known as IP-10 (IFN-γ-induced protein 10), is an important chemokine that belongs to the family of chemokines with a C-X-C motif (407). CXCL10 has been shown to be secreted by various cell types including macrophages, endothelial cells, fibroblasts and tumor cells (408). The expression of CXCL10 is significantly increased by the chemotaxis induced by IFN-γ and is involved in the chemotaxis of immune cells, the modulation of immune responses, and the immune surveillance of the TME (409).

#### 6.6.1 Role of CXCL10 in gastritis and GC

In chronic gastritis, CXCL10 enhances the immune response by recruiting CD4+ T cells and CD8+ T cells for infection control (341). CXCL10 modulates immune cell function and the intensity of local immune responses by binding to the CXCR3 receptor (410). CXCL10 potentiates the immune response against tumors and reduces tumor growth and metastasis, mainly by regulating immune cell migration and activation. The role of CXCL10 is to recruit immunosuppressive cells (such as Treg cells) to the tumor, and these cells suppress the activity of effector T cells (411).

#### 6.6.2 The role of CXCL10 in the TME

CXCL10, through its receptor CXCR3, plays a dual role in tumor immune escape (412). On the one hand, it recruits anti-tumor immune cells such as effector T cells and NK cells to the tumor site. This enhances the immune response and promotes tumor elimination (413). On the other hand, prolonged high expression of CXCL10 can lead to an overaccumulation of immunosuppressive cells, particularly Treg cells. Treg cells suppress effector T cell function and contribute to immune escape (414). Thus, its ability to direct immune cell recruitment, as well as the local immune status and cell types present, determine the impact of CXCL10 in the TME.

#### 6.6.3 The future of CXCL10

Because of its role in the modulation of immune responses, CXCL10 has emerged as a promising target for immunotherapy. Strategies that increase CXCL10 expression or activate its CXCR3 receptor could enhance anti-tumor immunity by promoting effector cell recruitment to the tumor site. The combination of CXCL10 modulation with immune checkpoint inhibitors (e.g. PD-1/PD-L1 inhibitors) (415), cancer vaccines or CAR T-cell therapies may improve overall therapeutic efficacy through synergistic enhancement of the immune response (416). As a result, the CXCL10/CXCR3 pathway is a valuable target for the development of novel immunotherapeutic strategies in cancers such as GC.

### 6.7 CX3CL1

CX3CL1, also known as fractalkine, is a unique chemokine. It belongs to the C-X3-C motif chemokine family (417). Unlike other chemokines, CX3CL1 can be expressed on the cell surface in either soluble or membrane-associated forms and plays important roles in the immune response, particularly in immune cell migration, inflammatory responses, tissue repair and the TME (418).

#### 6.7.1 Role of CX3CL1 in gastritis and GC

In gastritis, CX3CL1 regulates the migration of immune cells (particularly monocytes and macrophages) by binding to the CX3CR1 receptor and helps to direct immune cells toward the site of inflammation, thereby maintaining local immune responses and preventing the spread of pathogens (350). However, overexpression of CX3CL1 can lead to chronic inflammation that damages the lining of the stomach and increases the risk of GC, and can direct immunosuppressive cells, such as Treg cells, to accumulate at the site of inflammation, thereby supporting immune escape (419).

In GC, through increased recruitment of immune cells such as effector T cells and NK cells, CX3CL1 enhances the anti-tumor immune response and limits tumor growth and metastasis (352, 420).

#### 6.7.2 The role of CX3CL1 in the TME

By interacting with the CX3CR1 receptor, CX3CL1 recruits immunosuppressive cells (e.g., Treg cells, M2-type macrophages) to help tumors evade immune surveillance during immune escape in tumors (421, 422). At the same time, CX3CL1 enhances the secretion of immunosuppressive factors, inhibits the anti-tumor activity of effector T cells and NK cells, and promotes immune escape and tumor growth (423).

#### 6.7.3 The future of CX3CL1

By understanding the role of CX3CL1 in immune escape and tumor immune modulation, new targeted therapeutic strategies have been developed. In particular, new breakthroughs in the treatment of malignancies such as GC may be achieved through combination with immune checkpoint inhibitors, cytokine therapy and CAR T-cell therapy (424, 425).

## 7 Targeted agents against inflammatory cytokines

Targeted agents against inflammatory cytokines have been widely applied in various diseases, including hematological disorders, autoimmune diseases, and chronic inflammatory conditions, with their efficacy and safety well established (426–428). However, in inflammation-driven tumors—particularly in the context of GC—the therapeutic effectiveness and safety profile of these agents remain to be fully elucidated. The research progress of several targeted agents is summarized in **Table 6**. IL-6, TNF-α, and CXCL8 are three key pro-inflammatory cytokines extensively involved in remodeling the TME, thereby promoting tumor cell proliferation, metastasis, and immune evasion. Targeted interventions against these cytokines have entered preclinical or early-phase clinical research in various inflammation-associated diseases and selected malignancies, demonstrating considerable therapeutic potential.

**Table 6:** The application of inflammatory factor-targeted drugs in GC.

Drugs	Targets	Application status	Research in GC and gastritis
Canakinumab	IL-1β	FDA approved for CAPS, TRAPS, HIDS/MKD, FMF, AOSD, SJIA	NA
Anakinra	IL-1Ra	FDA approved for RA, DMARDs, NOMID, DIRA	NA
Gevokizumab	IL-1β	A phase III clinical trial (NCT02258867) for BD	NA
DFV890	IL-1β	A phase II clinical trial (NCT06031844) for CHD	NA
Aldesleukin	IL-2	FDA approved for RCC, Melanoma	NA
Basiliximab	IL-2Rα	FDA approved for AOR in patients receiving renal transplantation	NA
Bempegaldesleukin	IL-2	A phase II clinical trial (NCT03548467) for Melanoma, NSCLC	Preclinical research (429)
Nemvaleukin alfa	IL-2	A phase II clinical trial (NCT04144517) for HNSCC	NA
Dupilumab	IL-4Rα	FDA approved for AD, CRSwNP, Asthma, EoE	Preclinical research (430)
Tocilizumab	IL-6R	FDA approved for RA, CRS	NA
Siltuximab	IL-6	FDA approved for MCD (HIV/HHV-8 negative)	NA
Satralizumab	IL-6R	FDA approved for NMOSD (AQP4 antibody positive)	NA
Olokizumab	IL-6	A phase III clinical trial (NCT02760368) for RA	NA
Clazakizumab	IL-6	A phase II clinical trial (NCT03380377) for KTR	NA
CT-P47	IL-6R	A phase III clinical trial (NCT05489224) for RA	NA
Bazedoxifene	IL-6/IL-11/STAT3	A phase II clinical trial (NCT02448771) for BC	Preclinical research (431, 432)
BMS-986253	IL-8(CXCL8)	A phase II clinical trial (NCT02448771) for NSCLC, HCC	NA
Secukinumab	IL-17A	FDA approved for PsO, AS, PsA	Preclinical research (433)
Ixekizumab	IL-17A	FDA approved for PsO, PsA, AS	Preclinical research (433)
Sonelokimab	IL-17A/F	A phase II clinical trial (NCT05640245) for PsA	NA
Tadekinig alfa	IL-18BP	A phase II clinical trial (NCT02398435) for AoSD	NA
Ustekinumab	IL-12/IL-23p40	FDA approved for PsO, CD, UC	NA
Guselkumab	IL-23p19	FDA approved for PsO, PsA	NA
Risankizumab	IL-23p19	FDA approved for PsO, PsA, CD	NA
Tildrakizumab	IL-23p19	FDA approved for PsO	NA
Mirikizumab	IL-23p19	A phase III clinical trial (NCT05767021) for UC A phase III clinical trial (NCT04232553) for CD	NA
Infliximab	TNF-α/TNFR	FDA approved for RA, CD, UC, AS, PsO, PsA	Preclinical research (434)
Adalimumab	TNF-α	FDA approved for RA, CD, UC, AS, PsO, PsA	NA
Etanercept	TNF-α	FDA approved for RA, AS, PsA	NA
Golimumab	TNF-α	FDA approved for RA, UC, AS	NA
Certolizumab pegol	TNF-α	FDA approved for CD, RA, UC, AS	NA
Ozoralizumab	TNF-α	A phase III clinical trial (NCT04077567) for RA	NA
L19-TNF	TNF-α	A phase II clinical trial (NCT03420014) for STS	NA
Intron A	IFN-α2b	FDA approved for HCL, KS, CHB, CHC, Melanoma	Preclinical research (435)
Pegasys	IFN-α2a	FDA approved for CHB, CHC	NA
PegIntron	IFN-α2b	FDA approved for CHC, Melanoma	NA
Avonex	IFN-β1a	FDA approved for RRMS	NA
Rebif	IFN-β1a	FDA approved for RRMS	NA
Betaseron	IFN-β1b	FDA approved for RRMS, SPMS	NA
Actimmune	IFN-γ1b	FDA approved for CGD, Osteosclerosis	NA
Carlumab	CCL2	A phase II clinical trial (NCT00992186) for PCa	NA
Maraviroc	CCL5	FDA approved for HIV	Preclinical research (436, 437)
Leronlimab	CCL5	A phase II clinical trial (NCT01276236) for KS	NA
Reparixin	CXCL8/CXCR1/2	A phase II clinical trial (NCT01861054) for BC	Preclinical research (142, 438)
SX-682	CXCL8/CXCR1/2	A phase II clinical trial (NCT04599140) for CRC	NA
Vercirnon	CCL10/CCR9/CXCR3	A phase III (Terminated) clinical trial (NCT01536418) for CD	NA
Plerixafor	CXCL12/CXCR4/CXCR7	FDA approved for MM, NHL	Preclinical research (400, 439)
Motixafortide	CXCL12/CXCR4/CXCR7	FDA approved for MM	NA
Mavorixafor	CXCL12/CXCR4/CXCR7	FDA approved for WHIM Syndrome	NA
NOX-A12	CXCL12/CXCR4/CXCR7	A phase II clinical trial (NCT04121455) for GBM	NA

The information is sourced from https://clinicaltrials.gov/ and https://www.fda.gov/. NA, Not Applicable.

In the IL-6 signaling pathway, the IL-6 receptor antagonist Tocilizumab has been approved by the FDA for the treatment of rheumatoid arthritis and giant cell arteritis, and its potential application in solid tumors is gaining increasing attention. Related studies also indicate that Bempegaldesleukin, an IL-2 pathway agonist, significantly enhances the anti-tumor efficacy of radiotherapy through a T cell–dependent mechanism (429). Furthermore, Bazedoxifene inhibits IL-11–dependent STAT3 signaling, thereby blocking gastrointestinal tumor growth (431).

In the CXCL8 pathway, Reparixin, a CXCR1/2 receptor inhibitor, has been shown to markedly suppress the malignant behavior of GC MKN45 cells *in vitro* and *in vivo*. When combined with first- and second-line chemotherapy, it reduces toxicity and prolongs survival (438). Reparixin also diminishes the protective effect of CAFs on CD8^+^ T cells and improves the efficacy of anti-PD-L1 antibodies, thereby enhancing cytotoxic immune responses (142).

Plerixafor, a small-molecule CXCR4 antagonist, is a leading candidate in gastrointestinal cancer therapy targeting the CXCL12–CXCR4/CXCR7 axis (400). Studies demonstrate that Plerixafor modulates TAMs, suppresses GC progression, and enhances immune recognition and T cell activation (439).

In the TNF-α pathway, inhibitors such as Infliximab and Adalimumab are widely used in the clinical management of inflammatory bowel disease. Research suggests that Infliximab can suppress *H. pylori*–induced upregulation of CXCR4 by inhibiting TNF-α signaling, thereby reducing GC cell migration and exhibiting anti-tumor potential (434).

Additionally, the highly selective CCR5 antagonist Maraviroc, when combined with cisplatin, significantly inhibits the growth of GC organoids and shows promising anti- GC activity (436). Its mechanism may involve blocking the CCR5 pathway, thereby reducing GC cell migration induced by MIP-1α, MIP-1β, and RANTES (437).

Although the above targeted strategies have shown good safety profiles in approved disease settings, their application in the context of cancer still requires cautious evaluation. Inflammatory cytokines play essential roles in maintaining immune homeostasis; thus, long-term or systemic inhibition may lead to immune imbalance and an increased risk of infection. In addition, the presence of complex bidirectional regulatory mechanisms among different signaling pathways may result in unexpected immunosuppressive effects. In the future, it will be necessary to integrate tumor molecular subtypes, immune cell infiltration patterns, and peripheral pro-inflammatory cytokine levels to accurately identify patient populations most likely to benefit from cytokine-targeted therapies. A systematic assessment of the synergistic effects between cytokine inhibitors and immune checkpoint inhibitors, conventional chemotherapy, and anti-angiogenic therapies is needed to improve overall therapeutic efficacy and overcome resistance to monotherapy. With the aid of these technologies, the cellular sources and target sites of inflammatory cytokines can be precisely identified at single-cell resolution, thus providing a basis for individualized and precise therapeutic interventions.

## 8 miRNA-driven inflammatory persistence in gastric

MicroRNAs (miRNAs) regulate the intensity and persistence of inflammatory signaling by targeting multiple signaling components, acting as molecular adaptive mechanisms that facilitate immune evasion (440). In the context of *H. pylori* infection, key immunoregulatory miRNAs—particularly miR-155 and miR-146a—are significantly upregulated, thereby reprogramming TLR/NF-κB and associated downstream pathways (441). miR-155 is typically upregulated during infection and chronic inflammation, promoting or sustaining Th1/Th17 responses and functional remodeling of myeloid cells. However, its excessive or sustained expression may also indirectly promote immune evasion and pro-tumor microenvironment formation by modulating antigen presentation, suppressing certain inhibitory factors, or affecting immune checkpoint pathways. Conversely, miR-146a is often induced by NF-κB as a negative feedback regulator, targeting upstream adaptors like IRAK1/TRAF6 to reduce excessive inflammatory output and protect tissues (442). However, altered miR-146a expression (or functional imbalance) during chronic infection and carcinogenesis may contribute to dysregulated inflammation and influence tumor-associated NF-κB activity and cell proliferation signaling (443). Collectively, the dynamic regulation of miRNAs transforms pathogen-induced initial NF-κB/TLR signaling into a more persistent and individualized inflammatory state (444). This not only explains how inflammation-repair imbalance is sustained long-term to promote genomic instability and tumor progression but also reveals the value of miRNA regulatory axes as potential biomarkers or intervention targets.

## 9 Challenge and future perspective

In this review, we primarily focused on the inflammatory mechanisms underlying *H. pylori*–induced chronic gastritis and its progression to gastric cancer. However, relatively limited discussion was devoted to other well-defined etiologies of gastritis, such as autoimmune atrophic gastritis, bile reflux–related chemical injury, eosinophilic/lymphocytic or granulomatous gastritis, portal hypertensive gastropathy, and gastric mucosal injury caused by non–*H. pylori* infections (e.g., certain viruses or bacteria). Moreover, the prevalence of *H. pylori* infection varies across different geographic regions, which may influence the risk assessment and mechanistic understanding of gastric carcinogenesis. Future studies should place greater emphasis on the inflammatory characteristics of these distinct gastritis subtypes and their potential roles in gastric cancer development, thereby contributing to a more comprehensive understanding of the underlying pathogenic network.

### 9.1 CagPAI-mediated signaling cascades and pro-inflammatory responses

Among the various triggers of chronic gastritis, *H. pylori* infection represents the most well-characterized and potent inducer of gastric tumorigenesis. Persistent infection initiates and sustains mucosal inflammation through continuous activation of epithelial and immune signaling networks, ultimately transforming the gastric microenvironment into a pro-tumor niche. The cag pathogenicity island (cagPAI), a major virulence determinant, encodes the complete Cag type IV secretion system (Cag-T4SS) together with a set of structural and effector proteins that directly remodel host signaling at multiple levels (445, 446).

During intimate bacterial–epithelial contact, the Cag-T4SS assembles into transmembrane secretion and adhesion complexes, including the outer membrane core complex (OMCC) and sheath/axon-like structures (447). Structural components such as CagY, CagX, CagT, and CagM form the OMCC and determine the system’s material transport capacity, while effector proteins including CagA and the adhesion molecule CagL mediate host cell engagement and downstream signaling (448). CagL binds integrins (α5β1, αVβ6, etc.) with high affinity, activating the FAK/Src axis and receptor tyrosine kinase cascades (e.g., EGFR), leading to MAPK (ERK, JNK, p38) activation (449). This cascade induces AP-1 and NF-κB–dependent transcription of pro-inflammatory cytokines such as IL-8 and IL-6, establishing a strong chemokine gradient that recruits neutrophils and macrophages (450).

Concurrently, the Cag-T4SS delivers bacterial peptidoglycan (PGN) and CagA into host cytoplasm. Intracellular PGN is recognized by NOD1, triggering the canonical NF-κB and MAPK pathways that further amplify inflammatory gene expression (451). Once translocated, CagA undergoes phosphorylation at its EPIYA motifs by Src/Abl kinases; phosphorylated CagA aberrantly activates SHP2, leading to dysregulated growth factor signaling, enhanced proliferation, and motility (452, 453). Non-phosphorylated CagA binds the polarity regulator PAR1b, disrupting epithelial cell polarity and promoting epithelial–mesenchymal transition (EMT)-like changes (454). Additionally, CagA impairs DNA damage repair (e.g., BRCA1-dependent pathways), induces mitochondrial dysfunction and ROS accumulation, and increases genomic instability—all hallmarks of malignant transformation (455).

Chronic infection with cagPAI-positive *H. pylori* strains therefore promotes gastric carcinogenesis through sustained cytokine and chemokine secretion (IL-8, IL-6, TNF-α, IL-1β), which recruit and activate neutrophils and macrophages to produce reactive oxygen and nitrogen species (445). In parallel, persistent activation of IL-6/STAT3 and NF-κB signaling sustains epithelial survival and proliferation, while simultaneously inducing an immunomodulatory milieu characterized by the recruitment and polarization of MDSCs, regulatory T cells, and TAMs (456). These processes collectively establish a microenvironment with both pro-inflammatory and immunosuppressive features, fostering tumor initiation and progression.

With respect to inflammasome activation, studies suggest cell type– and strain-dependent variability. In macrophages and dendritic cells, H. pylori can “prime” NLRP3 via TLR2/NOD2 signaling, allowing pro–IL-1β synthesis and its Caspase-1–mediated maturation under specific stimuli. Conversely, other studies indicate weak or inhibitory effects on canonical NLRP3 activation, implying that H. pylori may fine-tune inflammasome responses to balance persistent inflammation and immune evasion (457).

### 9.2 HLA and inflammatory heterogeneity

HLA class I/II molecules form the core immunogenetic locus that regulates antigen presentation and determines the types of peptides presented to CD4+ and CD8+ T cells. This influences Th1/Th2/Th17 cell polarisation and the secretion of corresponding cytokine profiles (e.g. IFN-γ, IL-10, IL-1β and TNF-α) (458). Numerous studies have shown that the frequency of HLA-II alleles (particularly HLA-DQA1, HLA-DQB1 and HLA-DRB1) correlates with mucosal inflammation phenotypes and cytokine expression following H. pylori infection (459). In certain populations, specific HLA-II alleles have been found to correlate with either increased IL-10 expression or a heightened risk of pro-inflammatory factor production (e.g. IL-1β and TNF-α). This suggests that immunogenetic variation is a critical factor in explaining the differences observed in the intensity of the inflammatory response and disease susceptibility between individuals (460). Failing to consider HLA and antigen presentation polymorphisms restricts discussions of inflammatory responses to the ‘commonality’ level of pathogen-signalling pathways. This approach is unable to explain why different hosts exhibit markedly divergent inflammatory profiles and disease courses despite similar pathogen exposures.

### 9.3 Synergistic and antagonistic interactions of inflammatory cytokines and their signaling pathways in GC and gastritis

**Figure 2:**
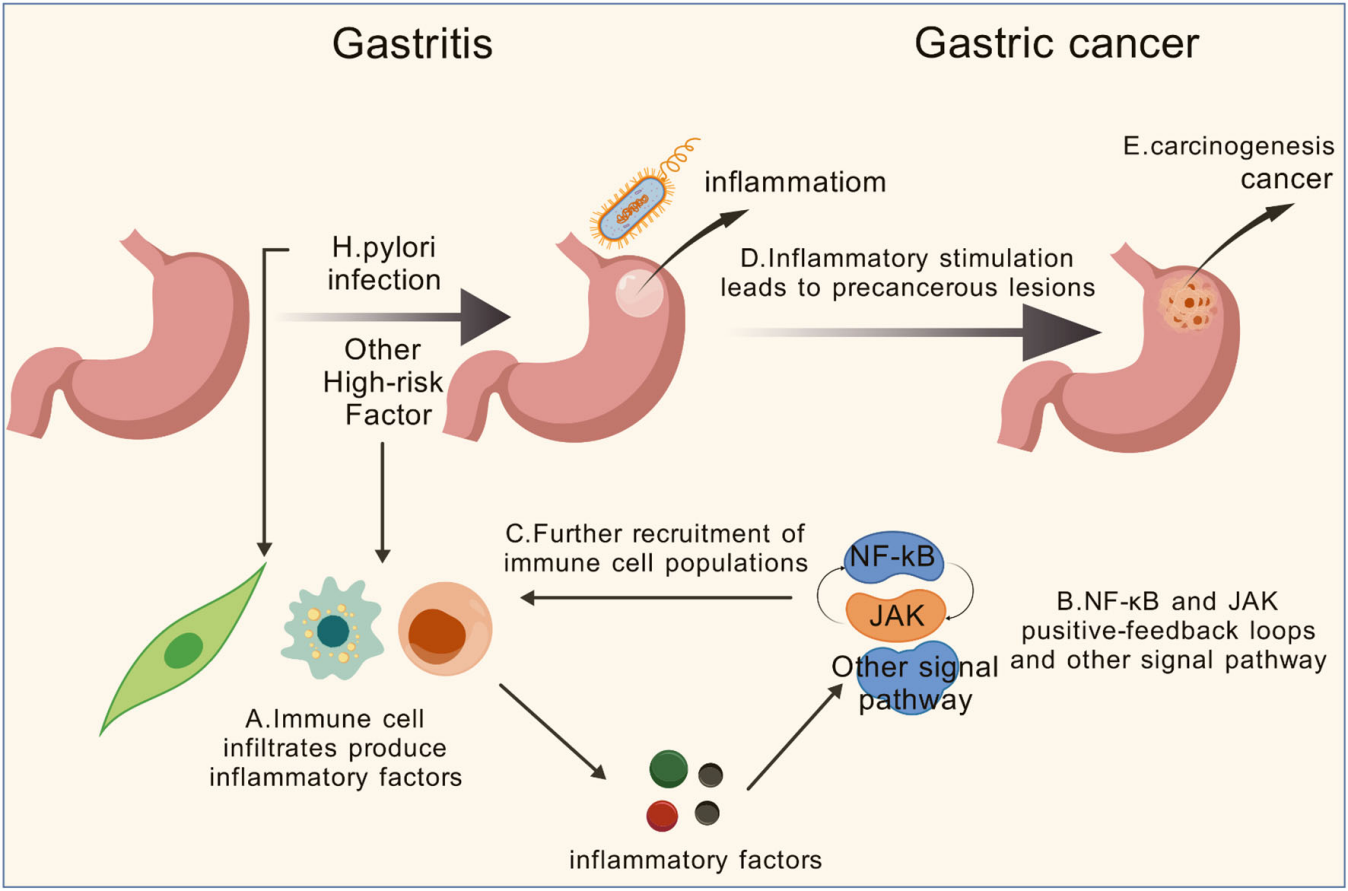
Progressive transition from chronic inflammation to GC: a multi-stage mechanism initiated by *H. pylori* infection and mediated by inflammatory signaling.

In the relationship between gastritis and GC, inflammatory factors play a crucial role (461, 462). A long-term chronic inflammatory response lays the foundation for the development of GC in chronic gastritis, especially that caused by *H. pylori* (463). The specific mechanisms of evolution are shown in **Figure 2**. This figure systematically illustrates how chronic gastric mucosal inflammation, induced by *H. pylori* infection or other high-risk factors, drives the progression from gastritis to GC. It highlights the cascade of inflammatory mediators and signaling pathways involved, along with their positive feedback regulation mechanisms.

Inflammatory factors (464) such as cytokines like IL-1, IL-6, TNF-α, IL-17 and chemokines like CXCL8 and CCL2 play an important role in this process. By activating multiple oncogenic signaling pathways (464) (e.g., NF-κB, JAK-STAT, MAPK, etc.), they promote tumor cell proliferation, survival, immune escape, and enhance tumor invasiveness and metastasis.

A. In the initial phase, *H. pylori* infection or other risk factors compromise the gastric mucosal barrier, leading to immune cell infiltration (e.g., neutrophils, macrophages, T cells). These cells release large amounts of pro-inflammatory cytokines such as TNF-α, IL-1β, IL-6, and CXCL8, marking the onset of the gastritis response.

B. In the second phase, inflammatory cytokines activate multiple signaling pathways, primarily NF-κB and JAK/STAT axes, which regulate immune amplification, cell survival, angiogenesis, and epithelial proliferation. These pathways engage in positive feedback loops that sustain and amplify the chronic inflammatory state. “Other signal pathways” may include MAPK, PI3K/AKT, and TLRs, which cross-regulate each other to enhance stress and injury responses in the gastric mucosa.

C. In the third phase, inflammatory mediators further promote immune cell recruitment and activation—e.g., CCL2-mediated monocyte/macrophage infiltration—forming a tripartite cycle of immune cells, cytokines, and signaling pathways that reinforce local inflammation.

D. In the fourth phase, sustained inflammation induces genetic mutations, stem cell damage, and epigenetic reprogramming in the gastric epithelium, leading to precancerous lesions such as intestinal metaplasia, atrophic gastritis, and dysplasia.

E. In the final phase, chronic inflammation promotes tumorigenesis by enhancing immune evasion, inducing EMT, and facilitating angiogenesis and stromal remodeling, ultimately driving the development of GC.

Inflammatory cytokines such as IL-1β, TNF-α, and IL-6 synergistically amplify immune responses during the early stage of gastritis via classical signaling pathways including NF-κB, JAK/STAT3, and MAPK, promoting mucosal hyperplasia, angiogenesis, and immune cell infiltration. Meanwhile, negative feedback regulators such as IL-10, TGF-β, SOCS3]/, and A20 maintain mucosal homeostasis by inhibiting these signaling axes and restrict excessive inflammation during the precancerous phase. However, when these antagonistic mechanisms become dysregulated or are hijacked by tumor cells, pro-inflammatory and pro-tumorigenic signals remain persistently active, while anti-inflammatory factors paradoxically facilitate immune evasion and microenvironment remodeling, thereby driving gastric carcinogenesis. This network exhibits marked heterogeneity both temporally (from early inflammation to precancerous lesions to advanced tumors) and spatially (across different mucosal regions and tumor core versus invasive margin). Only by constructing a multidimensional systems model integrating factors, pathways, disease stages, and spatial context can the dual regulatory roles and dynamic balance of inflammation in gastritis-to-GC progression be comprehensively elucidated. Detailed mechanisms are shown in **Tables 7** and **8**.

**Table 7:** Synergistic roles of inflammatory cytokines and their signaling pathways in GC and gastritis.

Factor/Pathway	Early-stage gastritis	Advanced-stage GC	Synergistic mechanism
IL-1β/NF-κB (465, 466)	*H. pylori* stimulate macrophages to secrete IL−1β, which activates NF−κB signaling in epithelial cells, leading to the release of chemokines and the recruitment of additional immune cells	In precancerous lesions, sustained activation of NF-κB by IL-1β promotes epithelial cell proliferation, angiogenesis, and ECM remodeling	IL-1β and NF-κB form a positive feedback loop, whereby NF-κB upregulates IL-1β expression, and IL-1β in turn further activates NF-κB
IL-6/JAK/STAT3 (133, 467)	Epithelial cells and infiltrating immune cells secrete IL-6, which activates STAT3 in epithelial cells, thereby promoting cell survival and regeneration.	The IL-6/STAT3 signaling pathway is highly expressed in GC, driving the maintenance of stem-like phenotypes and upregulation of immunosuppressive molecules	IL-6 and STAT3 form a positive feedback loop, wherein STAT3 upregulates the expression of IL-6 and its receptor, thereby enhancing signal persistence.
TNF-α/MAPK (291, 468)	Macrophages and activated T cells secrete TNF-α, which promotes activation of the p38 mitogen-activated protein kinase (p38/MAPK) pathway, thereby exacerbating mucosal injury and inflammation.	GC cells and TME macrophages co-secrete TNF-α, which enhances MAPK signaling to promote epithelial-mesenchymal transition and invasive potential.	TNF-α amplifies pro-inflammatory and pro-metastatic signals simultaneously through both NF-κB and p38 MAPK pathways, with these two pathways synergistically driving disease progression.
CCL2/CCR2 (355, 469)	CCL2 is upregulated at the site of inflammation, recruiting CCR2^+^ monocytes to migrate toward the mucosa.	TAMs secrete increased levels of CCL2 and cooperate with IL-10 and TGF-β to establish an immunosuppressive microenvironment.	CCL2 forms a network and other factors (JAK) to collaboratively recruit and activate pro-inflammatory and pro-tumor immune cells.

**Table 8:** Antagonistic roles of inflammatory cytokines and their signaling pathways in GC and gastritis.

Factor/Pathway	Early-stage gastritis	Advanced-stage GC	Synergistic mechanism
IL-10/STAT3 (144, 470)	IL-10 inhibits the activation of macrophages and dendritic cells, reducing the secretion of TNF-α, IL-1β, and IL-6, thereby alleviating mucosal inflammation.	In a subset of early-stage cases, IL-10 may restore CD8^+^ T cell function; however, its elevated expression in advanced stages can contribute to immunosuppression.	Activation of JAK/STAT and NF-κB signaling pathways promotes drug resistance in GC cells.
SOCS family (471, 472)	SOCS1 and SOCS3 are upregulated in response to stimulation by IL-6 and TNF-α, serving to limit excessive activation of the JAK/MAPK pathway and protect tissue integrity.	During the adenoma stage, SOCS3 is downregulated and inactivated, leading to sustained activation of STAT3; in advanced stages, it is further silenced through mechanisms such as CpG island methylation.	As a prototypical negative feedback inhibitor, it terminates signal transduction by directly binding to JAK or promoting receptor degradation.
A20 (TNFAIP3) (473, 474)	It is induced following NF-κB activation, reduces inflammatory signaling, and contributes to the maintenance of immune homeostasis.	In GC, A20 is frequently downregulated, resulting in sustained activation of NF-κB and promoting tumor progression.	By deubiquitinating EMT-related transcription factors, it ultimately leads to a malignant phenotype and poor prognosis of GC.

Immune cells such as Treg cells, MDSCs and M2-type macrophages infiltrate the TME and form an immunosuppressive microenvironment as the inflammatory response continues (475). These immunosuppressive cells inhibit an effective anti-tumor immune response through the secretion of immunosuppressive cytokines, thus allowing tumor cells to escape from immune surveillance (475). The development of immune escape mechanisms, which allow tumors to continue to grow under the pressure of the immune system, is an important feature of GC progression (476).

Inflammatory factors play an important role in immune escape in GC (477). Factors such as TNF-α and IL-1 exacerbate immune escape by promoting infiltration of immunosuppressive cells, upregulating immune checkpoint molecules such as PD-L1, and promoting tumor cell survival through pathways such as NF-κB (478–480). Thus, under the watchful eye of the immune system, GC cells can continue to grow and metastasize.

### 9.4 The neuroinflammation–tumor triangular interaction network

**Figure 3:**
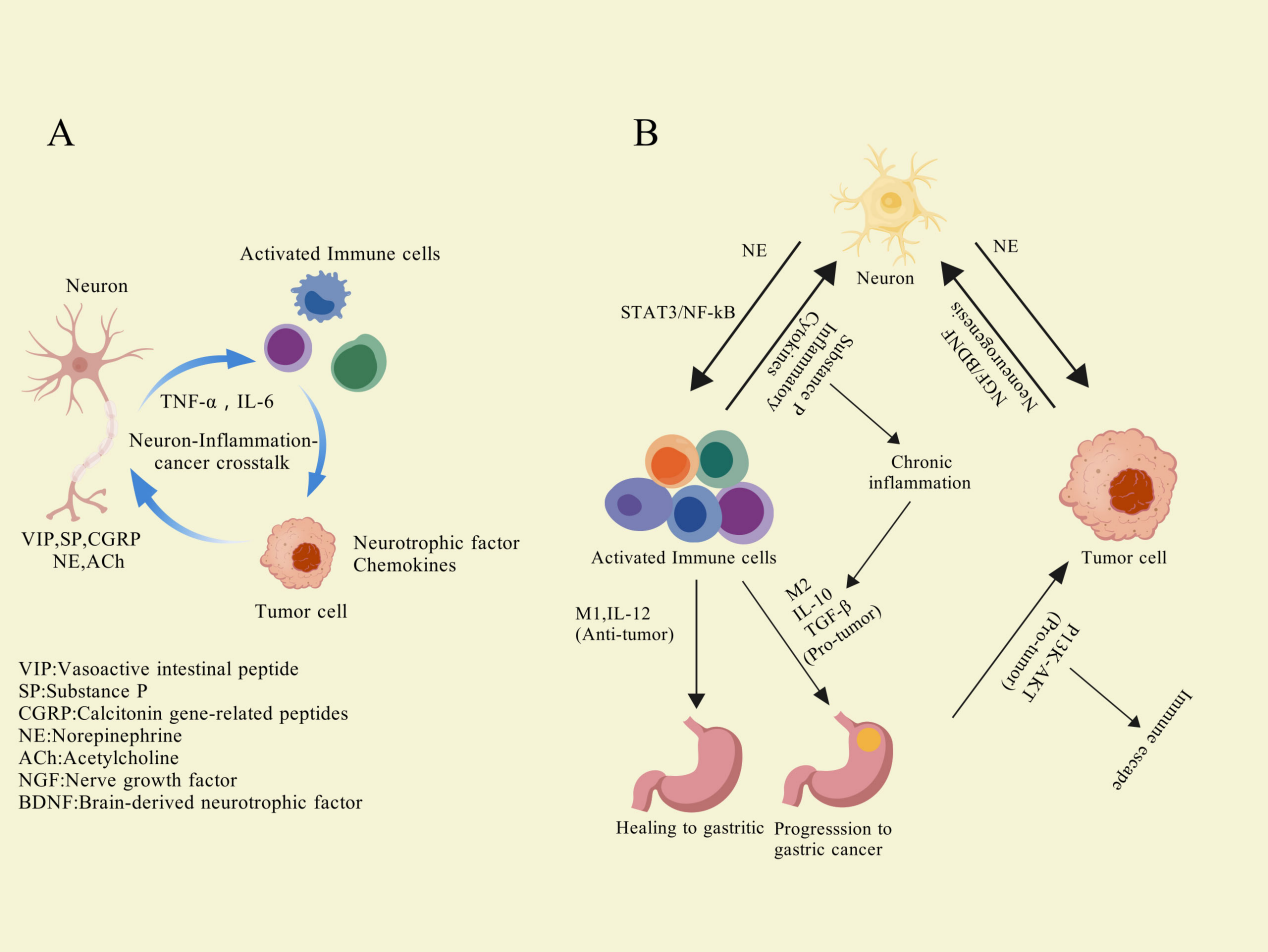
**(A)** Activated immune cells release pro-inflammatory cytokines such as TNF-α and IL-6, which enhance neuronal activity. The activated neurons then secrete neurotransmitters including VIP, SP, CGRP, NE, and ACh, which stimulate tumor cells to produce neurotrophic factors and chemokines. This reciprocal interaction sustains the neuro–inflammation–cancer signaling loop. **(B)** Relevant neurotransmitters promote an immunosuppressive microenvironment and tumor progression via the STAT3/NF-κB signaling pathway. This process facilitates the development of chronic inflammation and drives the polarization of immune cells from the M1 (anti-tumor) to the M2 (pro-tumor) phenotype, thereby shifting gastric tissue responses from inflammation repair toward gastric carcinogenesis. Meanwhile, tumor cells release neurotrophic factors that induce neural remodeling, further enhancing tumor growth and immune evasion.

Within the TME, the nervous system, immune inflammation, and tumor cells form a dynamically intertwined “third space” network. Neural signaling can regulate inflammatory responses, while inflammatory mediators, in turn, influence neuronal function. In parallel, both inflammation and neural activity jointly modulate tumor cell proliferation, migration, and invasion. Conversely, tumor cells can secrete various factors to remodel both the neural and immune landscape. These three components interact reciprocally and causally, constituting a “neuroinflammation–tumor” triangular interaction network (**Figure 3A**). The dysregulation of this network is a critical driving force behind tumor initiation, progression, and metastasis (372). Furthermore, the neuroimmune axis regulates immune responses through the vagus nerve and other neural pathways, maintaining immune homeostasis. This complex interplay acts as a double-edged sword in both inflammation and cancer. Inflammatory factors play a dual role in gastritis and GC, as shown in **Figure 3B**. Future research should focus on this crosstalk phenomenon, laying an important foundation for subsequent studies.

Within this network, the inflammatory response typically serves as the initiating event. Immune cells such as macrophages, dendritic cells, T cells, and microglia become activated within the TME and release a wide array of pro-inflammatory cytokines, including TNF-α, IL-1β, IL-6, and CXCL1. These cytokines not only directly promote tumor cell growth and metastasis but also act on local nerve endings, leading to increased neuronal excitability and neural remodeling.

Neural signaling regulates immune cells via adrenergic and cholinergic receptors. The sympathetic nervous system releases norepinephrine, which binds to β_2_-adrenergic receptors on macrophages, dendritic cells, and T cells, promoting M2 polarization and suppressing Th1 responses. This modulation influences cytokine production, cell migration, and overall immune function. Conversely, the parasympathetic nervous system regulates neural architecture through acetylcholine or modulates immune cell recruitment, polarization, and function via neuropeptides. Simultaneously, aberrant neural fiber growth within tumors—referred to as neoneurogenesis—can enhance tumor malignancy by transferring miRNAs and lncRNAs to tumor cells via exosomal pathways.

Tumor cells also play an active role in this interactive network. They can secrete neurotrophic factors (e.g., NGF, BDNF), chemokines (e.g., CXCL12), and extracellular vesicles to induce neural regeneration or remodeling, thereby establishing a more complex “tumor–nerve” axis. Some tumors even acquire neuronal-like properties through transcriptional reprogramming—a phenomenon known as neuronal mimicry—which enhances their responsiveness to neural signals. In addition, tumor-derived factors can reshape the inflammatory microenvironment by promoting the recruitment of immunosuppressive cells such as regulatory T cells and MDSCs, thus enabling immune evasion (475, 476).

This triangular interaction network can ultimately form a positive feedback loop: inflammation promotes neural activation; neural signals regulate immune responses; immune activity further facilitates tumor progression; and tumor cells, in turn, reactivate both inflammatory and neural pathways. Therefore, targeting the “neuro–inflammation–tumor” interaction network has emerged as a promising therapeutic strategy in cancer treatment. Potential approaches include blocking neurotransmitter signaling, inhibiting neurotrophic factors, modulating immune cell polarization, or applying denervation techniques to suppress tumor progression.

### 9.5 Application of emerging technologies

In recent years, emerging high-throughput technologies such as single-cell RNA sequencing (481, 482) and spatial transcriptomics (483, 484) have been widely applied in the study of gastrointestinal diseases, offering unprecedented resolution in elucidating the relationship between inflammatory factors and gastric pathologies. These techniques enable the dissection of transcriptional heterogeneity among different cell types—such as epithelial cells, immune cells, and fibroblasts—within the gastric mucosa at single-cell resolution, allowing for precise identification of the sources and targets of inflammatory mediators. For example, in models of chronic gastritis and *H. pylori* infection, single-cell analysis has revealed that pro-inflammatory cytokines such as IL-6 is primarily secreted by activated macrophages and mucosa-associated T cells, and can further influence the proliferation and differentiation trajectories of gastric epithelial stem cells (234, 485). Moreover, spatial transcriptomics enables the visualization of inflammatory factor expression across distinct anatomical regions of the gastric mucosa, thereby shedding light on the spatial relationship between localized inflammation and tumor progression. These advances are reshaping our understanding of gastric disease pathogenesis from the perspectives of cellular ecology and microenvironmental remodeling, and offer more precise strategies for early diagnosis and therapeutic intervention.

New avenues for the treatment of GC are emerging, including immunotherapy, particularly suppression of immune checkpoints such as PD-1/PD-L1 antibodies (486, 487), and targeted therapies against inflammatory factors (477, 488). Through the reversal of immune suppression and the reactivation of anti-tumor immune responses, these therapies are expected to be more effective in the treatment of GC patients. However, the challenge remains how to effectively control pro-inflammatory and escape mechanisms to improve patient prognosis.

## 10 Conclusions

This review highlights the central role of inflammatory factors in the transition from chronic gastritis to gastric cancer, emphasizing their interactions within the tumor microenvironment that promote both tumorigenesis and immune evasion. Inflammatory mediators establish a dynamic pro-tumor network through multiple signaling cascades. On one hand, they induce epithelial injury, stimulate aberrant proliferation, and foster genomic instability, thereby driving chronic inflammation toward malignant transformation. On the other hand, the same inflammatory signals sculpt an immunosuppressive microenvironment that dampens anti-tumor immunity and facilitates tumor immune escape. Thus, carcinogenesis and immune evasion represent interdependent processes—two facets of a single pathological continuum—linked by temporal and spatial feedback loops orchestrated by inflammatory signaling.

From this integrative perspective, targeting a single inflammatory pathway only offers limited and transient therapeutic benefit. A combinatorial strategy that suppresses pro-inflammatory signalling, reprograms immunosuppressive cells and activates anti-tumour immunity offers greater therapeutic potential. Biomarkers reflecting inflammatory network dynamics and the status of the TME are essential for patient stratification, combination therapy design and treatment response monitoring.

Furthermore, additional longitudinal clinical samples and mechanistic studies are required in order to identify biomarkers that can predict treatment response and guide stratified therapy. In summary, unravelling the interactive networks of inflammatory factors within the TME will provide a theoretical foundation for developing combined, personalised therapeutic strategies, ultimately improving clinical outcomes for gastric cancer patients.

## Glossary

GC: Gastric Cancer
*H. pylori*: Helicobacter pylori
IL: Interleukin
TNF: Tumor Necrosis Factor
IFN: Interferon
TME: Tumor microenvironment
ECM: Extracellular matrix
VEGF: Vascular endothelial growth factor
TAMs: Tumor-associated macrophages
MDSCs: Myeloid-derived suppressor cells
NSAIDs: Nonsteroidal anti-inflammatory drugs
TGF: Transforming Growth Factor
ROS: Reactive oxygen species
ADCs: Antibody-drug conjugates
PD-L1: Programmed death-ligand 1
PD-1: Programmed death-1
miRNAs: MicroRNAs
CagPAI: Cag pathogenicity island
Cag-T4SS: Cag type IV secretion system
PGN: Peptidoglycan
EMT: Epithelial–mesenchymal transition
CAFs: Cancer-associated fibroblasts
OMCC: Outer membrane core complex
TNFR: Tumor Necrosis Factor Receptor
MMPs: Matrix metalloproteinases
ISGs: Interferon-stimulated genes
MCP-1: Monocyte chemotactic protein-1
MIP-1α: Macrophage inflammatory protein-1α
RANTES: Regulated on Activation, Normal T Expressed and Secreted
MIP: Macrophage inflammatory proteins
SOCS: Suppressor of Cytokine Signaling
CAPS: Cryopyrin-Associated Periodic Syndromes
TRAPS: Tumor Necrosis Factor Receptor Associated Periodic Syndrome
HIDS: Hyperimmunoglobulin D Syndrome
MKD: Mevalonate Kinase Deficiency
FMF: Familial Mediterranean Fever
AOSD: Active Adult-Onset Still’s Disease
SJIA: Systemic Juvenile Idiopathic Arthritis
RA: Rheumatoid Arthritis
BD: Behçet’s Disease
CHD: Coronary Heart Disease
DMARDs: Disease modifying antirheumatic drugs
NOMID: Neonatal Onset Multi-System Inflammatory Disease
DIRA: Interleukin-1 Receptor Antagonist
RCC: Renal Cell Carcinoma
AOR: Acute organ rejection
NSCLC: Non-Small Cell Lung Cancer
HNSCC: Head and Neck Squamous Cell Carcinoma
AD: Atopic Dermatitis
CRSwNP: Chronic rhinosinusitis with nasal polyposis
EoE: Eosinophilic Esophagitis
CRS: Cytokine Release Syndrome
MCD: Multicentric Castleman Disease
NMOSD: Neuromyelitis Optica Spectrum Disorder
KTR: Kidney Transplant Rejection
BC: Breast Cancer
HCC: Hepatocellular Carcinoma
PsO: Psoriasis
AS: Ankylosing Spondylitis
PsA: Psoriatic Arthritis
AoSD: Adult -Onset Still’s Disease
CD: Crohn’s Disease
UC: Ulcerative Colitis
STS: Soft Tissue Sarcoma
HCL: Hairy Cell Leukemia
KS: Kaposi Sarcoma
CHB: Chronic Hepatitis B
CHC: Chronic Hepatitis C
RRMS: Relapsing Multiple Sclerosis
SPMS: Secondary Progressive Multiple Sclerosis
CGD: Chronic Granulomatous Disease
HIV: Human Immunodeficiency Virus
PCa: Prostate Cancer
CRC: Colorectal Cancer
MM: Multiple Myeloma
NHL: Non-Hodgkin Lymphoma
GBM: Glioblastoma
